# Dynamic regulation of myofibroblast phenotype in cellular senescence

**DOI:** 10.1111/acel.13580

**Published:** 2022-03-09

**Authors:** Irene López‐Antona, Constanza Contreras‐Jurado, Laura Luque‐Martín, Antonio Carpintero‐Leyva, Paula González‐Méndez, Ignacio Palmero

**Affiliations:** ^1^ Instituto de Investigaciones Biomédicas “Alberto Sols” CSIC‐UAM Madrid Spain; ^2^ Centro de Investigaciones Biológicas “Margarita Salas” CSIC Madrid Spain

**Keywords:** myofibroblast, plasticity, SASP, senescence, TGF‐β

## Abstract

Cellular senescence is an antiproliferative response with a critical role in the control of cellular balance in diverse physiological and pathological settings. Here, we set to study the impact of senescence on the regulation of cell plasticity, focusing on the regulation of the myofibroblastic phenotype in primary fibroblasts. Myofibroblasts are contractile, highly fibrogenic cells with key roles in wound healing and fibrosis. Using cellular models of fibroblast senescence, we find a consistent loss of myofibroblastic markers and functional features upon senescence implementation. This phenotype can be transmitted in a paracrine manner, most likely through soluble secreted factors. A dynamic transcriptomic analysis during paracrine senescence confirmed the non‐cell‐autonomous transmission of this phenotype. Moreover, gene expression data combined with pharmacological and genetic manipulations of the major SASP signaling pathways suggest that the changes in myofibroblast phenotype are mainly mediated by the Notch/TGF‐β axis, involving a dynamic switch in the TGF‐β pathway. Our results reveal a novel link between senescence and myofibroblastic differentiation with potential implications in the physiological and pathological functions of myofibroblasts.

## Graphical abstract

Primary fibroblasts under standard tissue culture conditions acquire myofibroblastic features. Cellular senescence results in the loss of myofibroblastic features in these cells. This phenotype can be transmitted by the senescent secretome, and it is associated with a dynamic switch in the TGF‐beta pathway during senescence. These results reveal a link between senescence and myofibroblastic differentiation with potential implications in the physiological and pathological function of myofibroblasts.

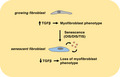

## 1 INTRODUCTION

Cell senescence is a stable form of cell cycle arrest linked to diverse pathological and physiological situations (Chan & Narita, 2019; Di Micco et al., 2021). The senescence program typically involves a blockade to proliferation followed by clearance mediated by immune cells, leading to the elimination of cells that undergo an unrepairable level of damage or are no longer required for normal tissue function. Senescence plays an essential role in the control of cell balance in diverse physiological settings such as embryonic development, wound healing, or regeneration. In turn, senescence dysfunction has been associated with a large number of diseases, in particular age‐related diseases, such as cancer, fibrosis, diabetes, and atherosclerosis (Munoz‐Espin & Serrano, 2014). Senescence is a complex, finely regulated cellular program that is connected with other essential cell processes. Among these, a link between senescence and cell plasticity has emerged recently, which appears to be markedly context‐dependent. For example, senescence can antagonize reprogramming to iPS in a cell‐autonomous manner (Banito et al., 2009) and diminish self‐renewal in aged adult stem cells (Sousa‐Victor et al., 2014). Paradoxically, senescence can also promote cell plasticity in vivo in a paracrine process mediated by the SASP (Chiche et al., 2017; Demaria et al., 2014; Mosteiro et al., 2016; Ritschka et al., 2017). In addition, the expression of a differentiation‐related gene signature is a typical feature in diverse senescence cellular models (Adrados et al., 2016; Storer et al., 2013). Here, we set to explore the link between senescence and cell plasticity, focusing on myofibroblastic differentiation. Myofibroblasts are a specialized cell type with fibroblastic and muscle cell features, which play key roles in several physiological and pathological contexts (Hinz et al., 2012). Myofibroblast function is required for tissue repair after injury, facilitating contraction and replenishing of extracellular matrix. The dysregulation of the physiological repair function of myofibroblasts can cause their aberrant accumulation leading to fibrosis that compromises organ function. While the origin of myofibroblasts in vivo is controversial, differentiation of resident fibroblasts is considered a major source of myofibroblasts (Hinz et al., 2012). Interestingly, previous studies have suggested a link of cell senescence to myofibroblast differentiation and fibrosis, but this relation seems complex and strongly context‐dependent (Schafer et al., 2018). For instance, the accumulation of senescent cells promotes lung fibrosis (Schafer et al., 2017), but an antifibrotic role of senescence has been described in liver or heart fibrosis (Krizhanovsky et al., 2008; Meyer et al., 2016) or during wound healing (Jun & Lau, 2010). In order to gain insights into the role of senescence in the differentiation and the physiological and pathological functions of myofibroblasts, here we have studied the regulation of myofibroblast phenotype in cellular models of fibroblast senescence. Our work shows that the myofibroblast phenotype is dynamically regulated during fibroblast senescence in a process that involves the senescence‐associated secretory phenotype (SASP), with differential regulation of the TGF‐β pathway.

## 2 RESULTS

### 2.1 Downregulation of myofibroblast phenotype during senescence

**FIGURE 1:**
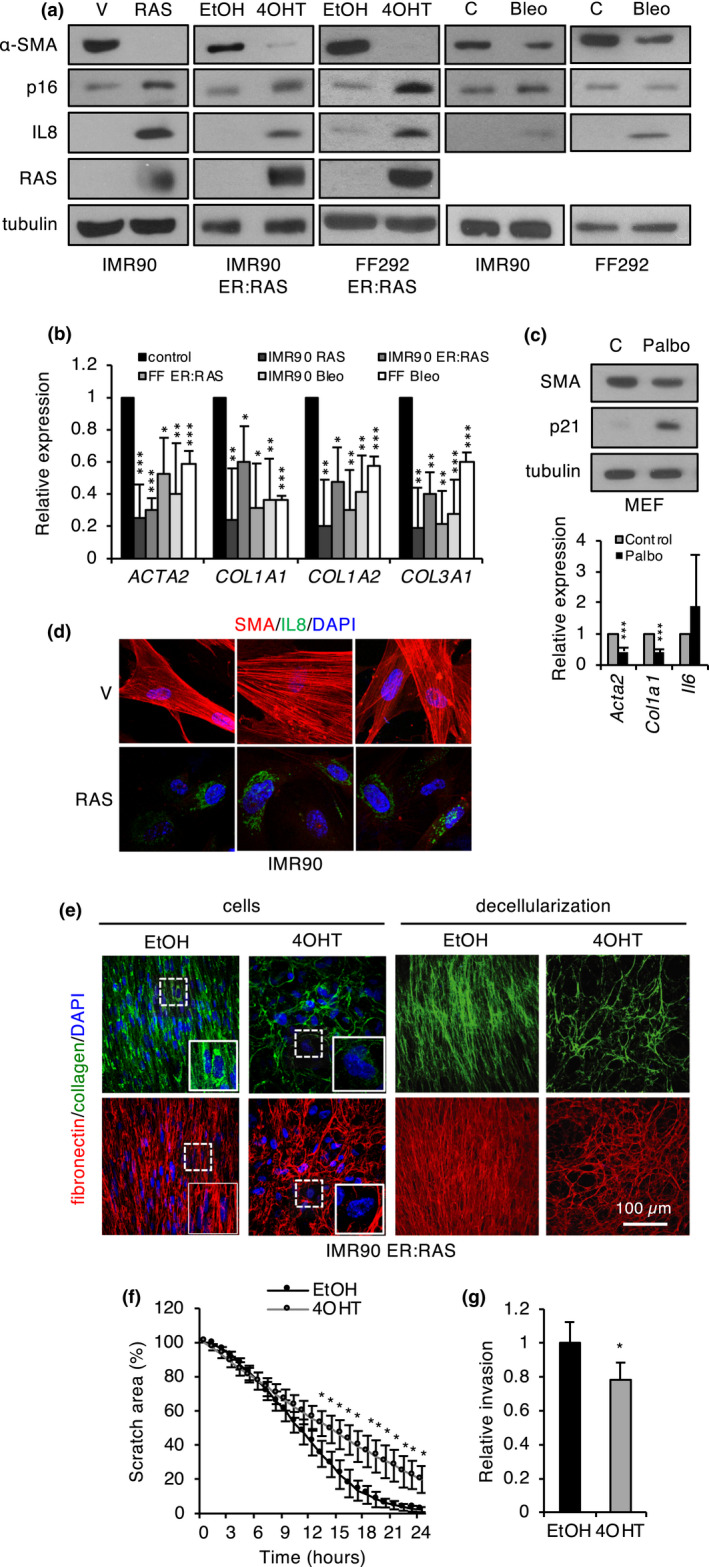
Regulation of myofibroblast markers in senescence. (a) Western blot analysis of α‐SMA, p16, IL‐8, and RAS/ER:RAS in the indicated human fibroblast cellular models of senescence. Tubulin was used as a loading control. (b) qPCR analysis of myofibroblast and ECM markers in the indicated cellular senescence models. The mean and standard deviation from at least three independent experiments are shown. (c) Western blot (top panel) and qPCR analysis (bottom panel) of the indicated senescence (p21), SASP (*IL*‐*6*), and myofibroblast (*Acta2*, *Col1a1*) markers in MEFs made senescent with palbociclib. The mean and standard deviation from three independent experiments are shown. (d) Immunofluorescence of α‐SMA, and IL‐8 in RAS‐senescent and control (V) human fibroblasts. (e) Immunofluorescence of collagen I and fibronectin 1 in senescent (4‐OHT) and control (EtOH) FF292 ER:RAS fibroblasts before (cells) or after decellularization. DAPI was used as a nuclear staining control. (f) Wound healing assay with senescent (4‐OHT) and control (EtOH) FF292 ER:RAS fibroblasts. The graph shows the scratch area at the indicated time points after scratching. The mean and standard deviation from three independent experiments are shown. (g) Invasion assay with senescent (4‐OHT) and control (EtOH) FF292 ER:RAS fibroblasts. The number of cells in the bottom of the membrane relative to the number of cells seeded was counted for each condition and then relativized to control fibroblasts. The mean and standard deviation from four independent experiments are shown. Bleo, bleomycin; Palbo, palbociclib

To investigate the impact of cell senescence on the regulation of the myofibroblastic phenotype, we analyzed the expression of myofibroblast markers in different cellular models of fibroblast senescence. Myofibroblasts are typically characterized by the expression of α‐smooth muscle actin (α‐SMA, *ACTA2*), associated with increased contractile force. They also show high expression of collagen genes, indicative of active deposition of extracellular matrix (ECM; Hinz et al., 2012). Notably, control non‐senescent fibroblasts showed detectable basal levels of α‐SMA, consistent with previous reports of expression of myofibroblast markers in fibroblasts in standard tissue culture conditions (Ehler et al., 1996). We found a consistent reduction in α‐SMA and collagen genes by Western blot and qPCR in two strains of primary human fibroblasts undergoing different forms of senescence, such as OIS (oncogene‐induced senescence) caused by chronic or inducible RAS expression, or DIS (DNA damage‐induced senescence) caused by bleomycin (Figure 1a,b, Figure S1a). Similar results were obtained in mouse embryo fibroblasts (MEFs) made senescent with the CDK inhibitor palbociclib (Figure 1c). Senescence induction was validated by reduced proliferation, increased SA‐BetaGal (senescence‐associated beta‐galactosidase) activity, and induction of the senescence effectors p16INK4A or p21CIP1, as well as IL‐8, a member of the inflammatory SASP (Acosta et al., 2008; Coppe et al., 2008) (Figure 1a,c and Figure S1b,c). Immunofluorescence analysis in the inducible ER:RAS model showed that α‐SMA was present in characteristic stress fibers and α‐SMA positivity showed a significant cell‐to‐cell inverse correlation with senescence markers such as the senescence‐associated heterochromatin foci (SAHF; Narita et al., 2003) and IL‐8 (Figure 1d), reinforcing the notion that myofibroblast markers are downregulated during senescence in fibroblasts. Of note, we did not detect a general correlation of α‐SMA levels with proliferation status (Figure S1d), suggesting that the observed phenotype was specifically associated with senescence.

Since myofibroblasts are associated with active ECM remodeling, we asked whether the observed changes in myofibroblast phenotype during senescence may have an impact in the ECM. To this end, we studied type I collagen and fibronectin by immunofluorescence in monolayers of growing and senescent ER:RAS fibroblasts, as well as in ECM after cell removal (Figure 1e). These stainings revealed clear changes in matrix patterns. The ECM from non‐senescent fibroblasts displayed a regular, compact pattern with parallel fibers, whereas senescent ECM showed a more irregular, lattice‐like pattern with large open spaces between fibers. This phenotype could be explained by the combined effect of altered matrix deposition and degradation mediated by matrix metalloproteases (Basisty et al., 2020; Birch & Gil, 2020). Of note, we also observed reduced protein levels of collagen and fibronectin in senescent fibroblasts (Figure 1e, left panel), consistent with the RNA expression data. The myofibroblastic phenotype is also linked to increased migration and invasion (De Wever et al., 2004). In vitro wound healing and Transwell Matrigel invasion assays revealed significantly reduced migration and invasion in OIS fibroblasts (Figure 1f,g). Collectively, these results indicate that the induction of senescence in cultured fibroblasts is accompanied by a loss of myofibroblastic phenotype.

### 2.2 The regulation of myofibroblast phenotype during senescence is mediated by the SASP

**FIGURE 2:**
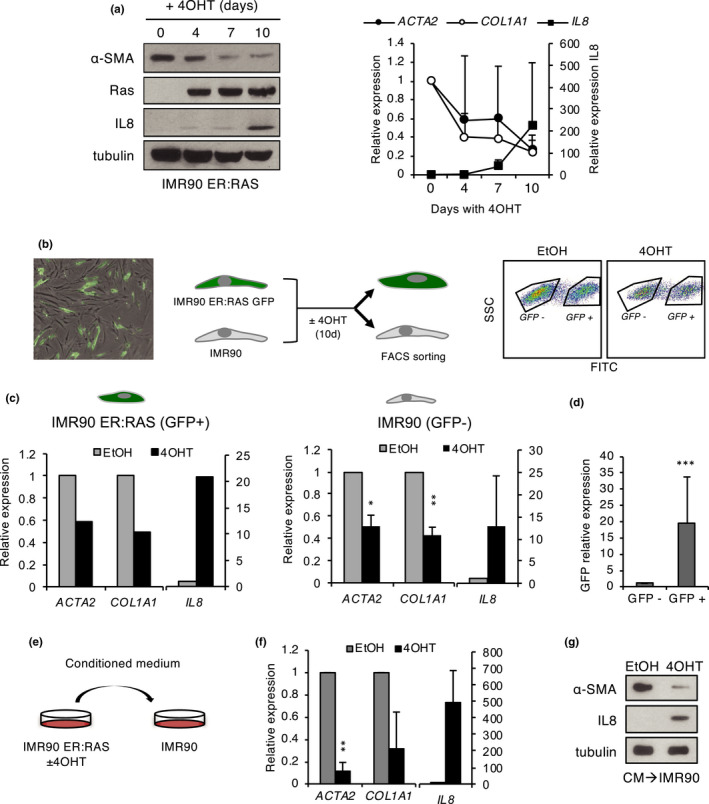
Paracrine transmission of myofibroblast phenotype regulation during senescence. (a) Western blot (left) and qPCR (right) analysis of *IL*‐*8* and the myofibroblast markers α‐SMA (*ACTA2*) and *COL1A1* during senescence induction with 4‐OHT in IMR90 ER:RAS fibroblasts. (b) Left panel: Representative image of co‐culture of IMR90 ER:RAS GFP and IMR90 fibroblasts (overlay bright field and GFP fluorescence) before adding 4‐OHT. Middle panel: experimental design of co‐culture experiments. Right panel: Flow cytometry sorting of GFP‐positive and GFP‐negative populations in co‐cultures treated with 4‐OHT or EtOH for 10 days. (c) qPCR analysis of *IL*‐*8* and myofibroblast markers in sorted GFP‐positive (IMR90 ER:RAS) and GFP‐negative (IMR90) populations. The mean and standard deviation from two independent experiments are shown for GFP‐negative cells; one representative experiment is shown for GFP‐positive cells. (d) qPCR of GFP in sorted GFP‐positive and GFP‐negative populations. (e) Experimental design of conditioned medium experiments. (f) qPCR analysis of *IL*‐*8* and myofibroblast markers in IMR90 fibroblasts exposed to conditioned medium from control (EtOH) and senescent (4‐OHT) IMR90 ER:RAS fibroblasts. The mean and standard deviation from two independent experiments are shown. (g) Western blot analysis of α‐SMA and IL‐8 in the same cells as in F

To further characterize the regulation of myofibroblast phenotype in senescence, we took advantage of the IMR90 ER:RAS‐inducible OIS model to examine the expression kinetics of myofibroblasts markers during senescence induction. Using Western blot and qPCR (Figure 2a), we observed a progressive decline in α‐SMA protein and transcript and *COL1A1* transcript during senescence implementation, which inversely correlated with the induction of IL‐8, a major component of the late‐inflammatory SASP (Acosta et al., 2008; Hoare et al., 2016). Similar expression kinetics were observed in FF292 ER:RAS fibroblasts (Figure S2a). To further investigate this apparent inverse correlation with the inflammatory SASP, we analyzed myofibroblast markers in two models where SASP production and senescence arrest could be dissociated. First, we used IMR90 shp53/shp16 fibroblasts as a model of SASP activation in the absence of senescent arrest. Upon RAS expression or ionizing radiation, IMR90 shp53/shp16 fibroblasts fail to implement a senescent cell cycle arrest (Figure S1b), but they retain a robust inflammatory SASP, as shown by a strong IL‐8 expression (Figure S2b; Coppe et al., 2008). Interestingly, myofibroblast markers displayed a significant downregulation in IMR90 shp53/shp16 cells with RAS or ionizing radiation, as assessed by Western blot, qPCR, and immunofluorescence (Figure S2b–d). Conversely, silencing of the senescence repressor SIX1 triggers p16INK4A‐mediated senescent arrest, without activation of the canonical inflammatory SASP (Adrados et al., 2016). Interestingly, in these cells, α‐SMA showed a small nonsignificant decrease, while *COL1A1* was upregulated, in a clear deviation from senescent fibroblasts with full SASP (Figure S2e–g).

Next, we asked whether this phenotype could be transmitted in a non‐cell‐autonomous manner, using two types of assays. First, IMR90 ER:RAS fibroblasts expressing GFP were co‐cultured with control, unlabeled IMR90 fibroblasts, and senescence was induced in the ER:RAS fibroblasts with tamoxifen. Then, both cell populations were separated using flow cytometry sorting based on GFP expression (Figure 2b,d). qPCR analysis of the sorted cell populations confirmed the downregulation of myofibroblast markers in OIS (GFP‐positive) fibroblasts. A significant reduction was also observed in normal (GFP‐negative) fibroblasts co‐cultured with OIS fibroblasts, relative to cells co‐cultured with control non‐senescent fibroblasts (Figure 2c). To determine whether this paracrine effect involved cell contact or rather whether it was mediated by soluble factors, we performed assays with conditioned medium. Non‐senescent IMR90 fibroblasts were incubated with medium conditioned by IMR90 ER:RAS fibroblasts with or without tamoxifen induction, and myofibroblast markers were examined by Western blot and qPCR in the recipient cells (Figure 2e). Normal fibroblasts exposed to conditioned medium from OIS cells, but not to control medium, showed a significant drop in myofibroblast markers, similar to that of senescent cells that coincided with a marked increase in the inflammatory SASP marker IL‐8 (Figure 2f,g). Taken together, these results clearly indicate that the senescent‐associated change in myofibroblast phenotype can be transmitted in a non‐cell‐autonomous manner, presumably via soluble SASP factors released to the medium.

### 2.3 Contribution of the fibrogenic and inflammatory phases of the SASP

**FIGURE 3:**
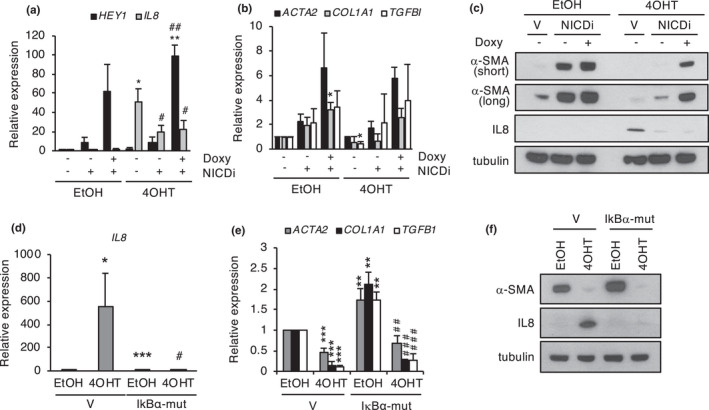
Role of the fibrogenic and the inflammatory SASPs in the regulation of myofibroblast phenotype. (a, b) qPCR analysis of the indicated genes in FF292 ER:RAS fibroblasts expressing inducible NICD (NICDi) or empty vector (V) treated with doxycycline in growing (EtOH) or senescence (4‐OHT) conditions. (c) Western blot analysis of α‐SMA (short and long exposures) and IL‐8 in the same conditions as in a and b. The mean and standard deviation from two independent infections are shown. (d, e) qPCR analysis of the indicated genes in FF292 ER:RAS fibroblasts expressing IκBα‐mut or empty vector (V). (f) Western blot analysis of α‐SMA and IL‐8 in the same cells as in d, e. The means and standard deviation from three independent experiments are shown. In a, the # symbol indicates significance relative to 4‐OHT/no Doxy/no NICDi; in e, the # symbol indicates significance relative to IκBα‐mut/EtOH

Recent evidence has shown that the composition of the SASP changes dynamically during senescence implementation, with a Notch/TGF‐β‐dependent fibrogenic SASP active at early time points, followed by an inflammatory SASP at later times (Hoare et al., 2016; Ito et al., 2017). Given the kinetics of myofibroblast marker expression during senescence and the link to the SASP shown above, we decided to study the role of the different waves of the SASP in the regulation of myofibroblast phenotype. To determine the contribution of the early Notch‐dependent SASP, we introduced in ER:RAS fibroblasts a doxycycline‐inducible form of the Notch1 intracellular domain (NICD), a product of proteolytic cleavage of membrane‐bound Notch that enters the nucleus to activate the Notch transcriptional program (Kopan & Ilagan, 2009). Doxycycline efficiently activated the Notch pathway, albeit with some leakiness, as indicated by the upregulation of Notch target genes such as *HEY1* and *HES1* (Figure 3a and data not shown). Notch pathway activation resulted in a dramatic increase in α‐SMA protein and transcript and, to a lesser extent, in *COL1A1* transcript, reverting the downregulation of these markers in senescent cells. As expected, IL‐8 protein and transcript were induced in senescent fibroblasts without NICD expression. Consistent with the reported crosstalk between the Notch fibrogenic SASP and the late‐inflammatory SASP (Hoare et al., 2016; Ito et al., 2017), IL‐8 levels were significantly reduced in NICD‐expressing senescent fibroblasts (Figure 3a–c). TGF‐β is a potent inducer of α‐SMA expression and myofibroblast phenotype, and it is also a downstream effector of Notch in the fibrogenic SASP (Hinz et al., 2012; Hoare et al., 2016). Consistent with the involvement of TGF‐β, we detected increased transcript levels of the TGF‐β target *TGFBI* in NICD‐expressing cells. These results suggest that the Notch/TGF‐β axis contributes to the regulation of myofibroblast phenotype during senescence. However, given the regulatory crosstalk between the fibrogenic and inflammatory SASPs (Hoare et al., 2016), these results would also be consistent with a potential involvement of the latter in this phenotype. To explore the role of the inflammatory SASP in this context, we used a mutant form of IκBα (IκBα mutant or super‐repressor) that acts as a constitutive repressor of the NF‐κB pathway (Boehm et al., 2007), a major transcriptional regulator of the inflammatory SASP (Acosta et al., 2008; Chien et al., 2011). As expected, the expression of IκBα mutant in ER:RAS fibroblasts efficiently blocked the induction of the inflammatory SASP marker IL‐8 in senescent cells (Figure 3d,f, Figure S3a,c). Interestingly, basal levels of myofibroblast markers were increased in non‐senescent cells expressing IκBα mutant. However, the downregulation of these markers in senescence was essentially unaffected, with kinetics indistinguishable from control cells (Figure 3e,f, Figure S3b,c). These results suggest that the senescence‐associated regulation of myofibroblast phenotype is independent of the NF‐κB‐regulated inflammatory SASP. In a complementary approach, we activated the NF‐κB pathway in non‐senescent cells using TNF‐α (Hayden & Ghosh, 2014). As expected, TNF‐α‐mediated activation of the NF‐κB pathway in non‐senescent cells resulted in a clear dose‐dependent induction of the SASP marker IL‐8, accompanied by a significant downregulation of myofibroblast markers (Figure S3d,e). Given the link between the inflammatory and fibrogenic waves of the SASP and the role of TGF‐β in myofibroblast differentiation, we wondered whether the effects observed upon modulation of the NF‐κB pathway in control cells could be linked to TGF‐β. Indeed, we found that the TGF‐β target *TGFBI* was significantly upregulated by the NF‐κB super‐repressor and downregulated by TNF‐α in control non‐senescent cells (Figure 3e and Figure S3d,e). This result suggests that the observed impact on myofibroblast markers under both conditions could, at least in part, be mediated by TGF‐β. This is consistent with previous reports of functional crosstalk between the NF‐κB and TGF‐β pathways (Freudlsperger et al., 2013). Consistent with the above findings, the combined manipulation of the Notch/TGF‐β and NF‐κB pathways in non‐senescent cells, using inducible NICD and TNF‐α, showed that TNF‐α strongly opposed the effects of NICD on *ACTA2* and *IL*‐*8* expression, at least in part, by inhibition of the TGF‐β pathway (Figure S3f). Collectively, this set of results suggests that the regulation of myofibroblast markers during senescence is mostly linked to the modulation of TGF‐β signaling, which may involve both the fibrogenic and inflammatory SASP.

### 2.4 Dynamic regulation of paracrine transmission of myofibroblast phenotype

**FIGURE 4:**
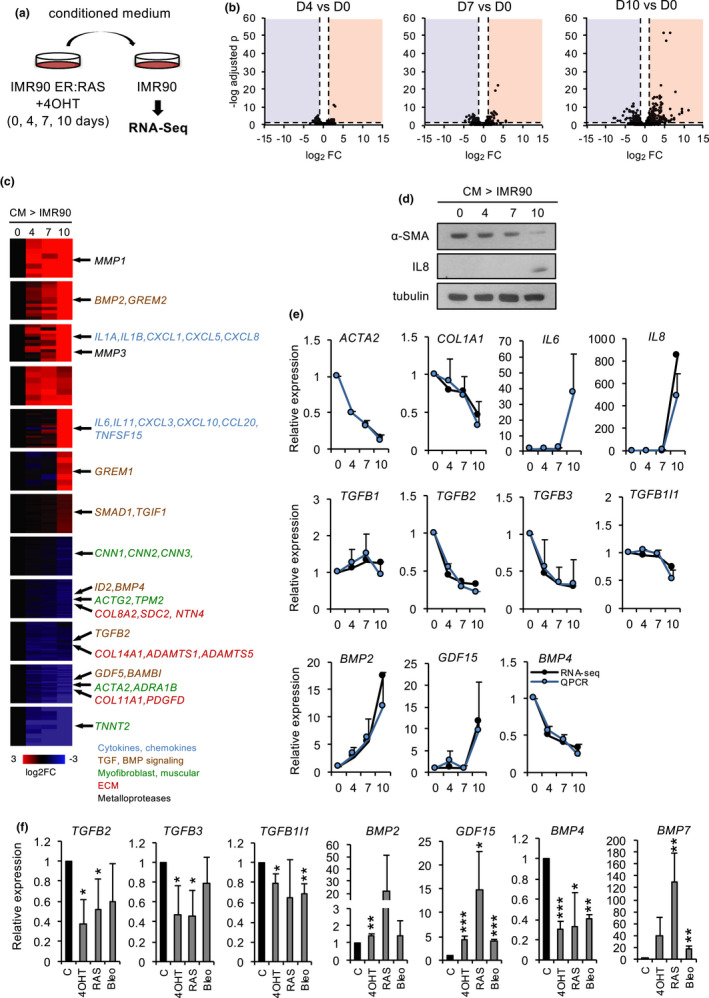
Transcriptomic dynamic analysis of paracrine senescence. (a) Experimental design. (b) *Volcano plot* of differential expression of D4, D7, and D10 cells relative to D0. The horizontal dashed line indicates *p *= 0.05, and the vertical dashed lines indicate fold change 2 or −2. (c) Expression clusters and representative genes. (d) Western blot of α‐SMA and IL‐8 in recipient fibroblasts along paracrine senescence kinetics. (e) Differential expression from RNA‐Seq and qPCR of the indicated genes in recipient fibroblasts along paracrine senescence kinetics. The mean and standard deviation from three independent experiments are shown for qPCR. (f) qPCR of the indicated genes in different forms of senescence in IMR90 fibroblasts. 4‐OHT: relative to EtOH control in ER:RAS cells; RAS, relative to vector‐infected cells; and Bleo, bleomycin‐treated cells relative to vehicle. The mean and standard deviation from three independent experiments are shown

To gain a more complete picture of the transmission of the senescence‐associated changes in myofibroblast phenotype, in connection with the SASP, we carried out a dynamic transcriptomic analysis of paracrine senescence using RNA‐Seq. To this end, normal IMR90 human fibroblasts were exposed to conditioned medium obtained from IMR90 ER:RAS fibroblasts at different time points (4, 7, and 10 days) after senescence induction with 4‐OHT, representative of the successive phases of the SASP (Figure 4a and Figure S4a). As expected (Acosta et al., 2013), exposure to late SASP induced a senescent phenotype in normal IMR90 fibroblasts (Figure S4b). For simplicity, normal fibroblasts exposed to medium obtained at day 0 post‐induction will be designated D0, and so on for the rest of samples (D4, D7, and D10). Volcano plots showed that the intensity of expression changes increases along the kinetics of senescence induction, with the most significant variations detected at D10, especially among upregulated genes (Figure 4b). Next, we sought to identify sets of genes with common expression patterns, using gene clustering (Figure 4c). A majority of clusters (13 out of 20 clusters, 227 genes) displayed increased expression along paracrine senescence, while 5 out of 20 clusters (183 genes) represented downregulated genes. Consistent with the volcano plot analysis, upregulated genes grouped in clusters with higher fold changes and sharper kinetics profiles, while downregulated gene clusters generally showed a more progressive kinetics with lower fold changes. The clustering analysis revealed distinct expression kinetic patterns in paracrine senescence (Figure 4c and Table S2). As expected, the most significantly enriched categories in upregulated clusters were related to the SASP and cell senescence (*senescence and autophagy in cancer*, *cytokine–cytokine receptor interaction*, and *NF*‐*κB signaling*), validating the experimental design. These categories included major components of the late‐inflammatory SASP, such *as IL*‐*1A*, *IL*‐*1B*, *IL*‐*6*, *IL*‐*8*, *CXCL1*, *and CXCL5*, as well as matrix metalloproteases such as *MMP1* and *MMP3* (Basisty et al., 2020; Coppe et al., 2008). Because of the expression kinetics of myofibroblast markers, next we focused on clusters with downregulated genes. Notably, this class was enriched in categories related to muscle phenotype and contraction, such as *vascular smooth muscle contraction*, *smooth muscle cell proliferation*, or *cytoskeleton*. These included the myofibroblast marker *ACTA2*, and related genes such as *ACTG2*, *TPM2*, and *TAGLN*. The categories related to extracellular matrix (*extracellular matrix organization*, *focal adhesion*) were also enriched in this set, including several collagens and fibrillin *FBN2*. The validation of the RNA‐Seq results by Western blot and qPCR confirmed the decreased expression of myofibroblast‐ and ECM‐related markers, as well as the upregulation of inflammatory SASP factors in paracrine senescence, with similar kinetics to autocrine senescence (Figure 4d,e). These results indicate gene expression changes related to myofibroblast phenotype and matrix dynamics during paracrine senescence. As such, they independently validate the notion of paracrine transmission of senescence‐associated changes in myofibroblast phenotype. Next, we interrogated the expression data to identify mechanisms involved in the regulation of this phenotype. Interestingly, we noted a significant enrichment in categories related to TGF‐β signaling pathway (*TGF*‐*β signaling pathway*, *signaling by BMP*) both in the upregulated and downregulated clusters (Figure 4c, Figure S4c). A detailed analysis of specific genes revealed an overall upregulation of the BMP branch (*BMP2*, *SMAD1*) and a repression of the TGF‐β branch (downregulation of *TGFB2*, upregulation of the inhibitor *TGIF1*) in paracrine senescence (Figure 4c). Of note, the downregulation of TGF‐β signaling suggested by the gene expression analysis was consistent with our above observations during manipulation of the SASP, and the known role of TGF‐β in regulation of myofibroblast phenotype.

### 2.5 Role of the TGF‐β pathway

Prompted by this evidence, we investigated further the role of TGF‐β signaling in this context. To do this, we analyzed in more detail the expression of members of this pathway in paracrine senescence and in different types of autocrine senescence (constitutive and inducible OIS and DIS). RNA‐Seq results and qPCR (Figure 4e,f) showed that the TGF‐β2 (*TGFB2*) and TGF‐β3 (*TGFB3*) ligands, but not TGF‐β1 *(TGFB1)*, as well as the *TGFB1I1* target gene, were significantly downregulated in autocrine and paracrine senescent fibroblasts. In contrast, members of the BMP branch, such as *BMP2*, *BMP7*, but not *BMP4*, were significantly upregulated, as was the TGF‐β distantly related factor *GDF15* (Figure 4e,f). Moreover, we used an antibody array to detect secreted TGF‐β pathway factors in the conditioned medium of senescent ER:RAS fibroblasts. This semiquantitative assay confirmed the reduced levels of members of the TGF‐β branch, such as TGF‐β2 and TGFBI, and the increased levels of members of the BMP branch, such as BMP2 and BMP7, in senescent conditioned medium. Interestingly, for a large fraction of factors, the effect was retained in IκBα mutant fibroblasts, indicating that this regulation was, at least in part, independent of the NF‐κB‐regulated inflammatory SASP (Figure S4d). Collectively, these results confirm the paracrine transmission of the senescence‐associated changes in myofibroblast phenotype and suggest that these changes may be associated with the differential regulation of the BMP and TGF‐β branches of the TGF‐β superfamily.

**FIGURE 5:**
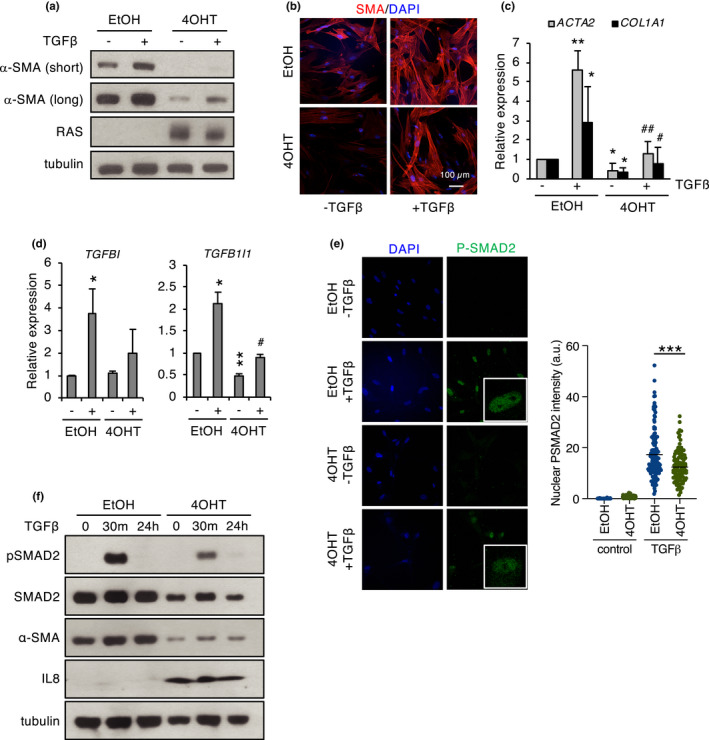
TGF‐β signaling in senescence. (a) Western blot analysis of α‐SMA (short and long exposures) in IMR90 ER:RAS fibroblasts in growing (EtOH) or senescence (4‐OHT) conditions after treatment with TGF‐β (2 ng/ml, 48 h). (b) Immunofluorescence of α‐SMA in the cells described in (a) DAPI was used as a control nuclear stain. (c) qPCR of the indicated myofibroblast markers in the cells described in (a). (d) qPCR of the indicated TGF‐β target genes in the cells described in (a). (e) Immunofluorescence of phospho‐SMAD2/3 in FF292 ER:RAS fibroblasts in growing (EtOH) or senescence (4‐OHT) conditions treated with 10 ng/ml TGF‐β for 30 min. DAPI was used as a control nuclear stain (left). Quantification of nuclear phospho‐SMAD2 intensity (right). The # symbol indicates significance relative to 4‐OHT/control. (f) Western blot analysis of phospho‐SMAD2 in FF292 ER:RAS fibroblasts in growing (EtOH) or senescence (4‐OHT) conditions treated with 10 ng/ml TGF‐β for the indicated times and untreated controls

We next sought to determine whether the cellular response to TGF‐β was affected by senescence. To this end, control and senescent fibroblasts were treated with TGF‐β. As expected, ectopic TGF‐β efficiently induced myofibroblast markers in growing fibroblasts (Hinz et al., 2012); however, this effect was significantly reduced in senescent ER:RAS cells (Figure 5a–c). Similar results were obtained in OIS fibroblasts due to constitutive RAS expression, and in IMR90 shp53/p16 RAS fibroblasts (SASP induction without senescent arrest) (Figure S5a,b). Additional TGF‐β‐inducible genes, such as *TGFBI* and *TGFB1I1*, also showed blunted induction by TGF‐β in ER:RAS‐senescent fibroblasts, consistent with defective TGF‐β signaling in senescence (Figure 5d). Of note, treatment with TGF‐β alone did not cause significant induction of senescence markers (Figure S7a). The phosphorylation and nuclear translocation of the SMAD2/3 transcription factors are well‐established readouts of active TGF‐β signaling. Using immunoblot and immunofluorescence, we observed that the total protein levels, phosphorylation, and nuclear accumulation of SMAD2 in response to TGF‐β were reduced in OIS fibroblasts relative to non‐senescent controls (Figure 5e,f, Figure S6). These results further support the notion that TGF‐β signaling is impaired in our cellular model of senescence (Figure 5e,f) providing a potential mechanism for the observed downregulation of myofibroblast phenotype in fibroblast senescence.

## 3 DISCUSSION

Cell senescence is a complex antiproliferative program involved in a variety of physiological and pathological situations (Chan & Narita, 2019; Di Micco et al., 2021). Here, we have used cellular models of senescence to study the impact of senescence in the regulation of the myofibroblastic phenotype in fibroblasts. This process has important biological implications, given the essential role of myofibroblasts in key physiological and pathological settings, such as wound healing or fibrosis (Hinz et al., 2012). In our studies, we have taken advantage of the fact that primary fibroblasts in standard tissue culture conditions display features of the canonical myofibroblast phenotype. These include the expression of key myofibroblast markers, such as α‐SMA, contractile properties, and increased invasion and migration (Ehler et al., 1996). Our data clearly show that the implementation of different forms of senescence consistently leads to the repression of markers and functional features characteristic of myofibroblasts. Mechanistically, our co‐culture and conditioned medium assays indicate that the senescence‐associated changes in myofibroblast phenotype can be transmitted in a paracrine manner. Although a role for extracellular vesicles cannot be formally excluded, our data are consistent with transmission through soluble SASP factors. Recent results have identified the existence of successive waves of SASP along senescence induction (Hoare et al., 2016; Ito et al., 2017). Interestingly, the downregulation of myofibroblast markers in senescence follows a kinetics that is reminiscent of this temporal shift in SASP composition. Our results from the transcriptomic analysis of paracrine senescence and manipulation of signaling pathways collectively suggest that the senescence‐associated changes in myofibroblast phenotype are most likely caused by the temporal changes in the TGF‐β pathway observed during senescence. Notably, there is evidence that the two main branches of the TGF‐β pathway can have antagonistic effects in myofibroblast differentiation and fibrosis. It is well established than the TGF‐β branch is a potent inducer of myofibroblast differentiation with strong profibrotic action (Hinz et al., 2012; Zent & Guo, 2018). Thus, the reduced TGF‐β activity in late senescence reported here could clearly contribute to blunting the myofibroblast phenotype. Conversely, members of the BMP branch have been shown to be antifibrotic, including BMP2 and BMP7, upregulated during senescence in our study (Dituri et al., 2019). At this stage, the potential involvement of the BMP branch in control of the myofibroblast phenotype is less clear. Preliminary experiments with recombinant BMP2 and BMP7 in control fibroblasts showed a pro‐senescence effect, in line with previous reports (Acosta et al., 2013; Kaneda et al., 2011). However, we have not been able to detect a significant impact of individual BMP factors in myofibroblast marker expression, at least in the conditions used (Figure S7b). Further studies are clearly warranted to clarify the role of the BMP branch in this context.

The communication of the senescent cell with its environment, including the ECM, is an emerging feature of the senescent phenotype (Fafian‐Labora & O'Loghlen, 2020). It has been proposed that interactions between cellular and matrix proteins can regulate senescence (Hiebert et al., 2018; Rapisarda et al., 2017). Our results identify a potential novel layer of regulation of the senescent phenotype related to the crosstalk with the ECM. Mechanical stress from the cell microenvironment is recognized as a major determinant of the myofibroblast phenotype. Our data, in line with previous reports (Hiebert et al., 2018; Mavrogonatou et al., 2017; Mellone et al., 2016), clearly indicate that senescent cells may influence the composition and, presumably, physical properties of the ECM. Moreover, the storage of latent TGF‐β or other signaling molecules in the matrix could be similarly affected by senescence‐specific changes in the ECM. Thus, it is conceivable that the senescent ECM may provide mechanical and chemical signals critical for the modulation of myofibroblastic traits, and perhaps other features of the senescent phenotype. Previous studies indicate that the link of senescence to myofibroblast differentiation and fibrosis is complex and strongly context‐dependent (Schafer et al., 2018). On the one hand, senescence may act as a mechanism limiting fibrosis in liver, heart, and wound healing (Jun & Lau, 2010; Krizhanovsky et al., 2008; Meyer et al., 2016). On the other hand, it has also been proposed that senescence can promote fibrosis in other settings, including lung, kidney, and pancreas fibrotic diseases (Kellogg et al., 2021; Schafer et al., 2017). Our results clearly show the downregulation of myofibroblast markers in cellular models of senescence. Despite differences between our in vitro model and the physiological context, these observations might reflect an alternative mechanism by which senescence could limit fibrosis. In addition to the canonical antiproliferative effect of senescence, which would restrain the expansion of myofibroblasts (Krizhanovsky et al., 2008), our data suggest that senescence could also lead to the reversal of the specialized myofibroblast phenotype, which may synergistically contribute to resolution of fibrosis (Jun & Lau, 2018). Interestingly, a similar differentiation‐limiting effect of senescence as the one shown here has been described for muscle differentiation in fibroblasts (Latella et al., 2017). Such a role for senescence in modulating differentiation is in line with previous reports linking senescence to cell plasticity in different contexts (Chiche et al., 2017; Mosteiro et al., 2016; Ritschka et al., 2017) and highlights the complex crosstalk of senescence with cell plasticity regulation. In summary, our results unveil a novel link between cellular senescence and myofibroblastic differentiation, associated with dynamic changes in the TGF‐β pathway. These findings may have important implications in the role of senescence in the function of this cell type in physiology and disease.

### 3.1 Limitations of the study

In this report, we have used different cellular models of fibroblast senescence to address the connection between senescence and specification of the myofibroblast phenotype. Cellular models of senescence have been extensively used and have proved instrumental to decipher basic mechanisms in senescence. However, these in vitro models do not accurately reflect the complexity of pathophysiological situations associated with myofibroblast function in live organisms. Further in vivo studies would be informative to determine the impact of our findings in the context of physiological or pathological responses mediated by myofibroblasts.

## 4 EXPERIMENTAL PROCEDURES

### 4.1 Cell culture and treatments

IMR90 human embryo lung primary fibroblasts were obtained from ECACC. FF292 human adult primary dermal fibroblasts were a gift from Marisol Soengas, CNIO, Madrid. IMR90 ER:RAS fibroblasts (expressing the ER:RAS cassette) have been previously described (Adrados et al., 2016), FF292 ER:RAS fibroblasts were generated with the same strategy. Mouse embryo fibroblasts were obtained from E13.5 embryos. All the cells were cultured in Dulbecco’s modified Eagle’s medium (DMEM; Gibco) with 10% fetal bovine serum (FBS; Gibco), containing penicillin and streptomycin, and were used at low passage. To induce senescence, cells were treated with palbociclib (HY‐A0065; MedChemExpress) at 10 μM for 9 days or bleomycin (bleomycin sulfate; Mylan) at 10 µM for 24 h and analyzed 6 days later. Fibroblasts were also irradiated with 10 Gy and analyzed 10 days later. Doxycycline (D1822; Sigma) was used at 1 µM. TNF‐α (300‐01A; PeproTech) was used at 10 and 20 ng/ml for 48 h. Human BMP2 protein (355‐BM‐010/CF; R&D Systems) was used at 50 μg/ml for 24 h. Human BMP7 protein (354‐BP‐010/CF; R&D Systems) was used at 400 μg/ml for 24 h. For TGF‐β treatments, cells were grown in DMEM with 0.01% FBS for 48 h prior to the addition of TGF‐β1 (100‐21; PeproTech).

### 4.2 Senescence‐associated beta‐galactosidase staining

Cells were plated at a density of 3 × 10^4^ cells per well in six‐well plates. The following day, they were fixed and stained as described (Adrados et al., 2016).

### 4.3 Viral transduction

Retroviral and lentiviral transductions were performed as described (Adrados et al., 2016). The following vectors were used: pQCXIH‐i‐N1ICD‐Flag (a gift from Masashi Narita, CRUK, Cambridge), pBabe‐IκBα‐mut (Addgene #15291), and pRRL‐CMV‐IRES‐EGFP (a gift from Antonio Bernad, CNB, Madrid). Other vectors used in this study have been described elsewhere (Adrados et al., 2016).

### 4.4 In vitro scratch assay

FF292 ER:RAS cells treated with 4‐OHT or vehicle for 7 days were seeded in six‐well plates (2 × 10^5^ cells/well). Two days later, scratches were performed with a sterile tip. Then, complete DMEM was replaced by DMEM with 1% FBS. Gap closure was analyzed in a Cell Observer microscope (Zeiss), taking photographs every hour during 24 h. Images were analyzed with ImageJ MRI Wound Healing Tool.

### 4.5 Invasion assay

FF292 ER:RAS cells treated with vehicle or 4‐OHT for 7 days were incubated with DMEM with 1% FBS for 24 h. Matrigel (356231, BD) solution (12 μg in 100 μl of FBS‐free DMEM) was added to a Transwell (3422; Corning) and incubated overnight at room temperature. Then, complete DMEM was added to the bottom chamber, and 4 × 10^3^ cells were seeded on each Transwell. After 24 h, bottom membranes were fixed with 4% paraformaldehyde for 15 min and stained with DAPI (1:500, D1306; Invitrogen), and nuclei were counted in a Nikon 90i fluorescence microscope.

### 4.6 Co‐culture experiments

IMR90 ER:RAS/GFP cells (expressing pRRL‐CMV‐IRES‐EGFP) were mixed with parental IMR90 in a 1:2 ratio. Ten days after addition of 4‐OHT or vehicle, cells were sorted based on GFP expression using flow cytometry (FACSVantage SE; Becton Dickinson). Total RNA was extracted from each population using miRNeasy Mini Kit (217004; Qiagen) and analyzed by qPCR as described.

### 4.7 Immunofluorescence

Cells were plated on glass coverslips in six‐well dishes (1.5 × 10^5^ cells/well). The day after, they were fixed with 4% paraformaldehyde for 20 min, permeabilized with 0.1% Triton X‐100 for 15 min, blocked with 2% BSA or 5% serum from the same species as the secondary antibody, and incubated overnight with primary antibodies (see Table S1). Except for primary antibodies coupled to fluorochrome, cells were then incubated with fluorochrome‐conjugated secondary antibodies for 2 h at room temperature in the dark. Finally, cells were mounted in Prolong with 1:500 DAPI (D1306; Invitrogen). Images were captured in a confocal microscope (LSM710; Zeiss).

### 4.8 EdU incorporation

Cells were plated on glass coverslips in six‐well dishes (1.5 × 10^5^ cells/well). The day after, EdU was added at 10 µM for 6 h (IMR90), 60 min (FF292), or 90 min (MEFs). Then, cells were fixed with 4% paraformaldehyde (Merck) for 15 min, washed twice with 3% bovine serum albumin, permeabilized with 0.1% Triton X‐100 for 20 min and washed again. Finally, EdU was detected using Click‐iT EdU Alexa Fluor 647 Imaging Kit (C10340; Molecular Probes). Samples were analyzed in a Nikon 90i fluorescence microscope, counting at least 100 cells for each condition.

### 4.9 Conditioned medium assays

Conditioned medium assays were performed essentially as described in Adrados et al. (2016). DMEM serum‐free medium was added to cells for 72 h, harvested, filtered and stored at −80 until use. Incubation with recipient cells was done for 96 h, after addition of FBS to a final concentration of 1%.

### 4.10 Extracellular matrix assays

Glass coverslips were treated with 0.2% gelatin in PBS for 1 h at 37°C, washed with PBS, and treated with 1% glutaraldehyde (49630; Fluka) for 30 min at room temperature. After washing, 1 M ethanolamine (E9608; Sigma) was added for 30 min at room temperature. After washing, control or senescent fibroblasts were seeded (10^5^ cells per 24‐well dish). At days 1, 3, and 5, DMEM containing 50 µg/ml ascorbic acid was added. At day 7, cells were left untreated or removed adding decellularization buffer (20 mM NH_4_OH, 0.5% Triton X‐100) for 15 min at room temperature. After three washes with PBS, cells were processed for immunofluorescence as described above.

### 4.11 Western blot

Protein lysates were prepared with medium salt buffer (150 mM NaCl, 50 mM Tris, pH 8.0, 1% NP‐40) with protease (cOmplete, 11836170001; Merck) and phosphatase (PhosSTOP, 04906845001, Merck) inhibitors. SDS‐PAGE and Western blot were performed as described in Adrados et al. (2016) using 15 µg of protein per lane, unless otherwise specified. The primary and secondary antibodies used are described in Table S1.

### 4.12 RNA extraction and quantitative PCR

Total RNA was extracted with TRI Reagent (AM9738; Thermo Fisher Scientific) as described (Gomez‐Cabello et al., 2010). RT‐PCR was performed with the Applied Biosystems 7900HT PCR System using SYBR Green, at the Genomics Service of the Instituto de Investigaciones Biomédicas (Madrid). 18s ribosomal RNA was used as a reference. Primer sequences are shown in Table S1.

### 4.13 RNA sequencing

Normal IMR90 fibroblasts were incubated for 3 days with conditioned medium obtained from IMR90 ER:RAS fibroblasts at days 0, 4, 7, and 10 of induction with 4‐OHT. Total RNA from two independent experiments (RNA integrity number between 9.5 and 10) was used for RNA sequencing at the Genomics Unit of CNIO (Madrid), essentially as described (De Lope et al., 2019). Differential expression was calculated for each time point (days 4, 7, and 10) relative to day 0, using DESeq2. Clustering of genes with similar expression kinetics was performed using MeV (Multiple Experiment Viewer) with genes with significant (*p *< 0.05) differential expression in at least one time point. Twenty clusters were built with *K*‐means clustering using Euclidean distance. Two clusters with only one gene were discarded and the rest used for further study. Functional enrichment was done with Enrichr software using the following libraries: Transcription, Pathways, and Ontologies. Heatmaps were built with the Heatmapper tool.

### 4.14 Antibody array

Conditioned medium of FF292 ER:RAS fibroblasts with or without overexpression of IκB‐α mutant in growing (EtOH) or senescent conditions (4‐OHT) was analyzed with RayBio^®^ C‐Series Human TGF Beta Array 2 (AAH‐TGFB‐2‐4; RayBiotech) following the manufacturer´s recommendations. Signal intensity was measured with Fiji.

### 4.15 Statistical analysis

The graphs show the mean, standard deviation, and statistical significance. Statistical significance was calculated using Student’s *t* test: *p < 0.05, **p < 0.01, and ***p < 0.001.

## CONFLICT OF INTEREST

The authors declare no conflicting interests.

## AUTHORS’ CONTRIBUTIONS

IL‐A, CC‐G, LL‐M, AC‐L, and PG‐M performed experiments. IP supervised the project, secured funding, and wrote the manuscript with input from all the authors.

## Supporting information

Fig S1‐S8

Table S1

Table S2

## Data Availability

The RNA‐Seq data generated in this study have been deposited at Gene Expression Omnibus (GEO) with the accession number GSE139563 (López‐Antona & Palmero, 2019).
