# Supplementary material for: Dynamic regulation of myofibroblast phenotype in cellular senescence
Source: Aging Cell. 2022 Mar 9;21(4):e13580. doi: 10.1111/acel.13580 (PMC9009235; doi:10.1111/acel.13580)

Suppl Figure 1

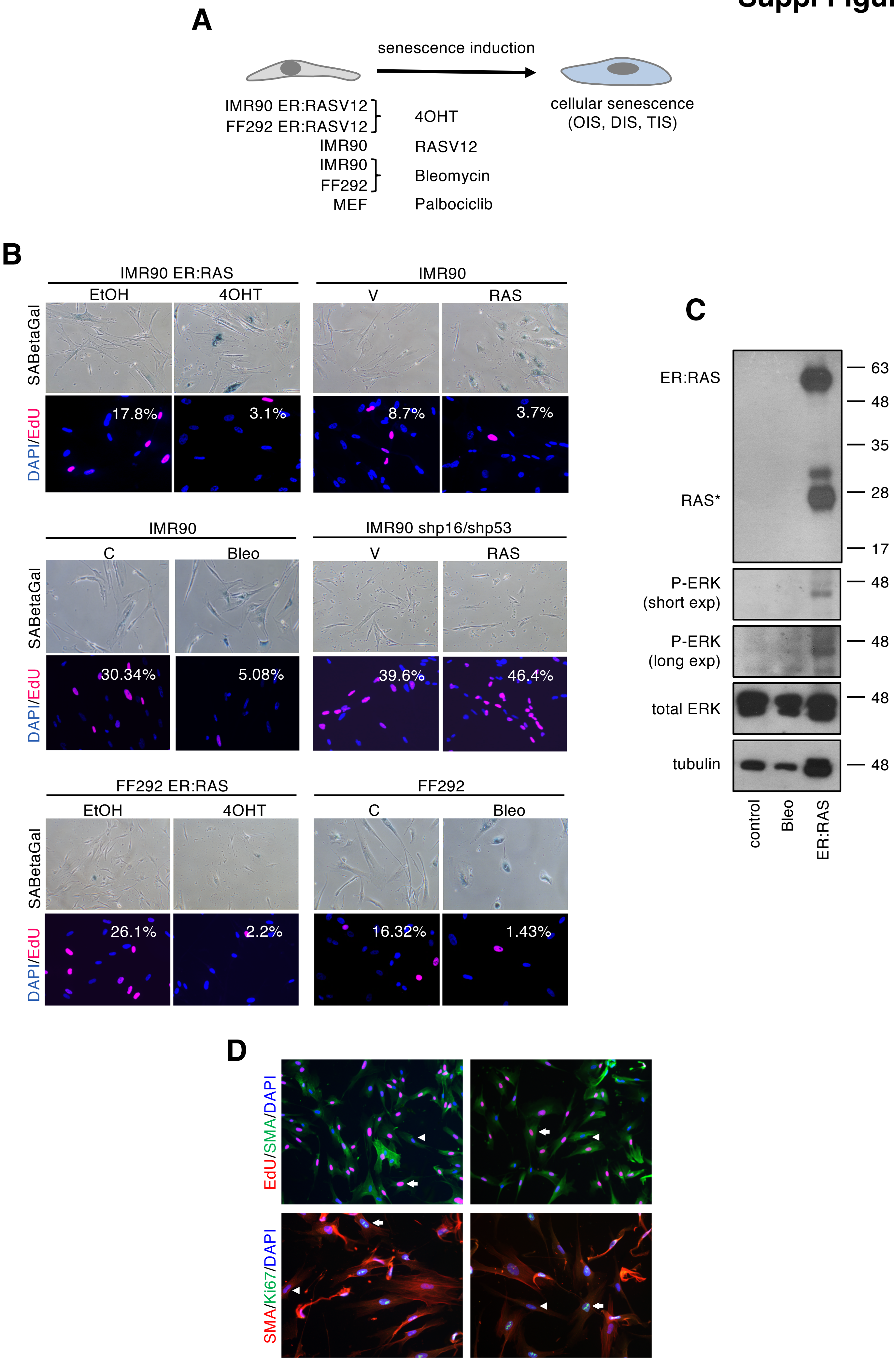

Suppl Figure 2

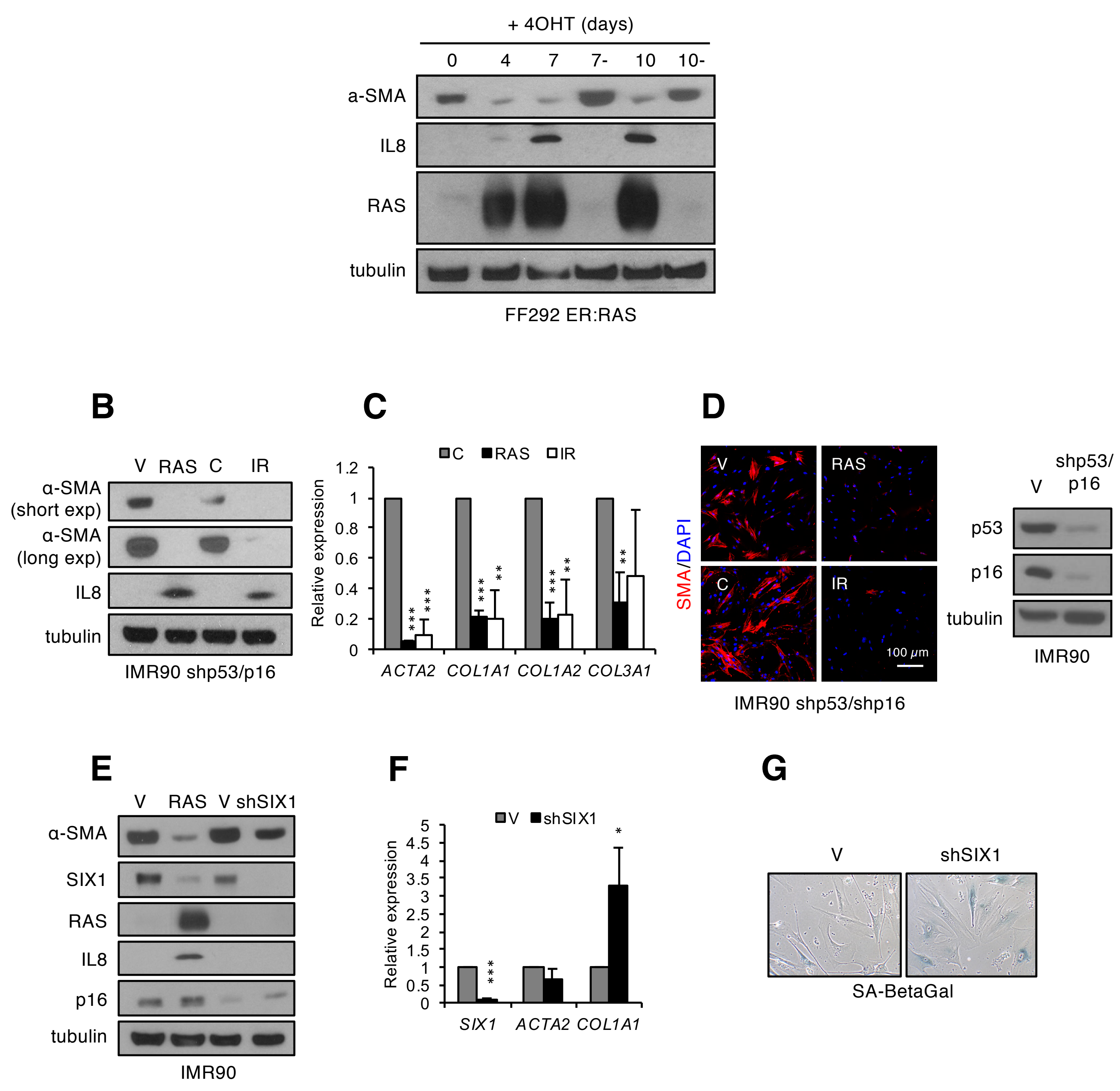

Suppl Figure 3

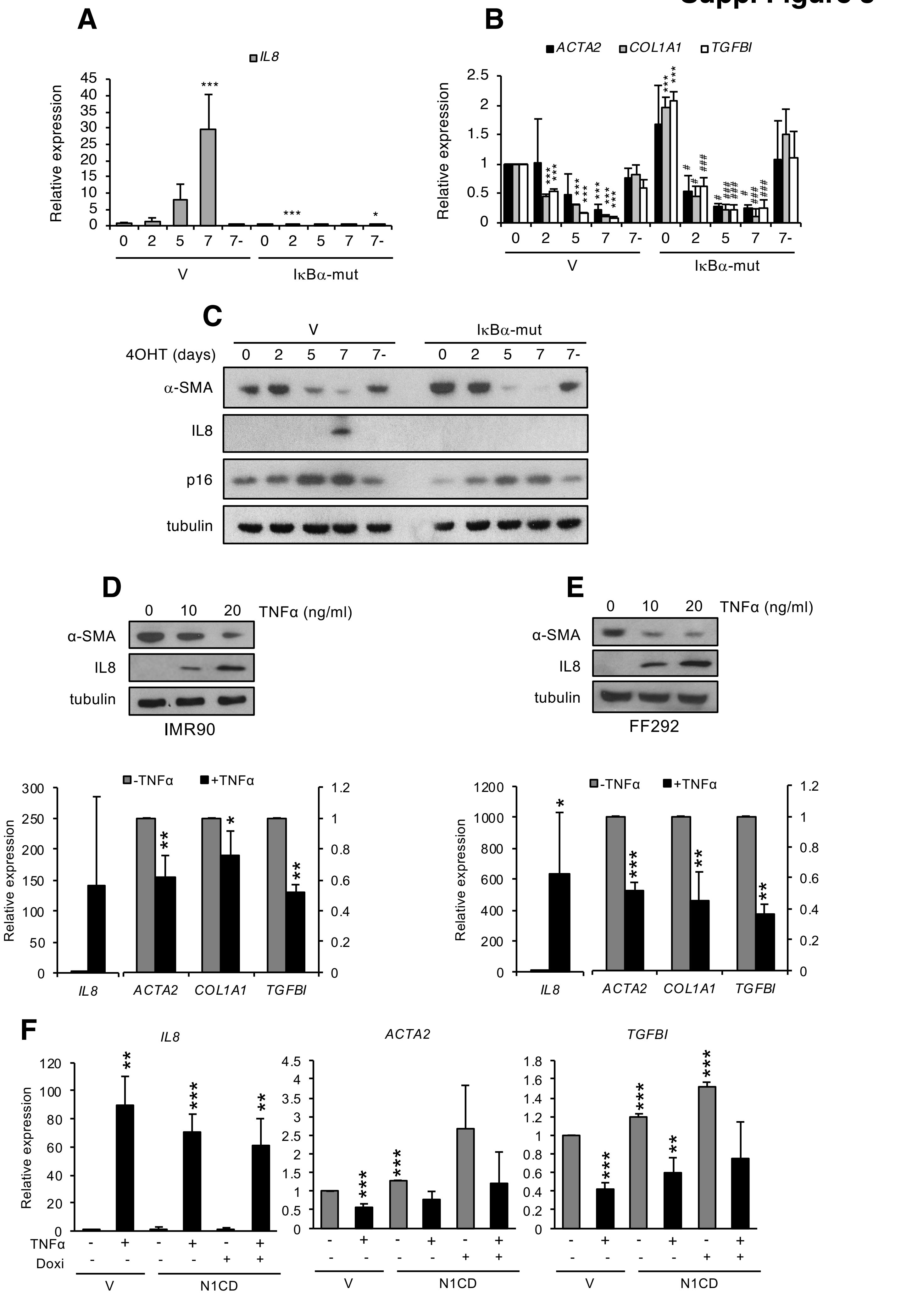

Suppl Figure 4

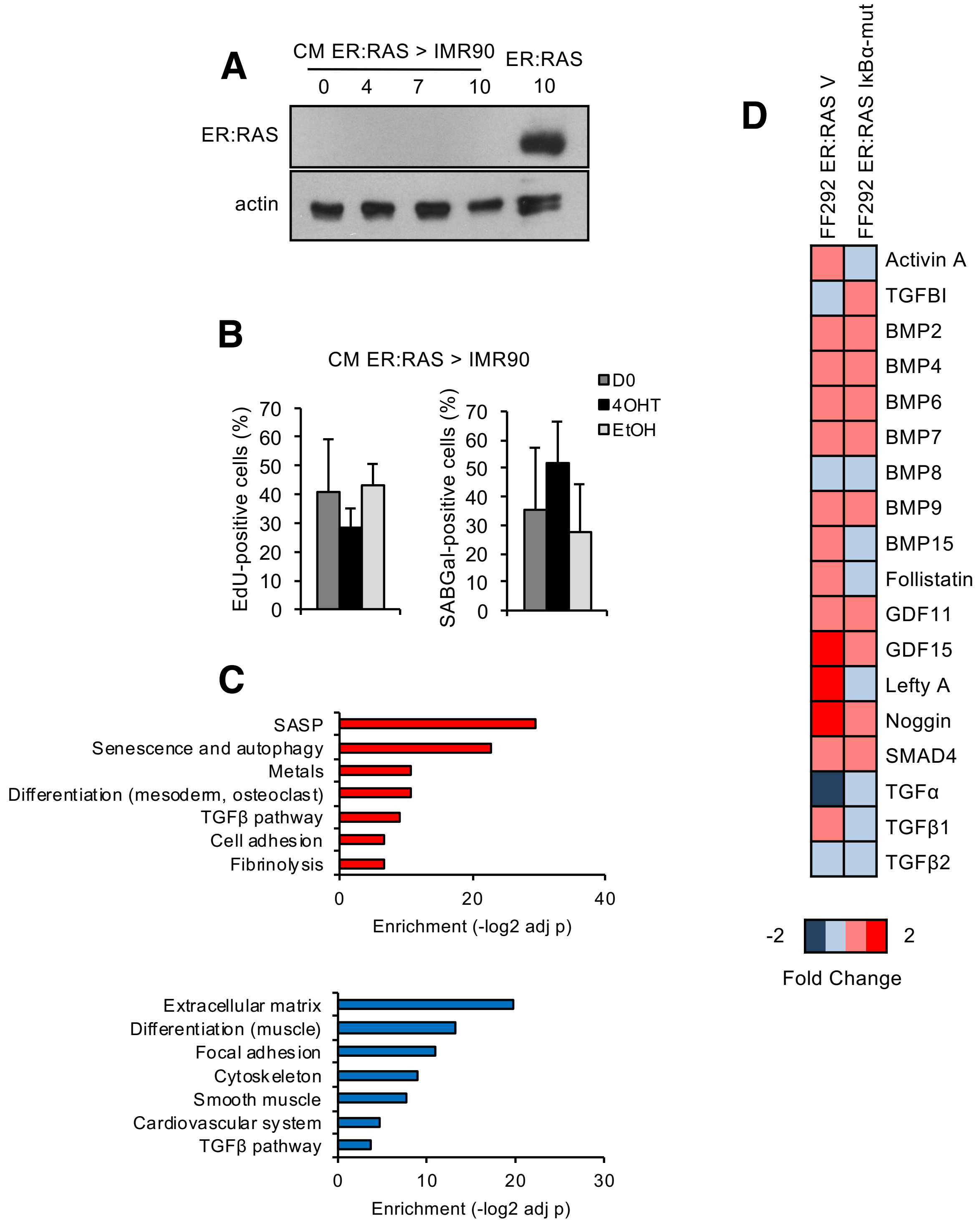

Suppl Figure 5

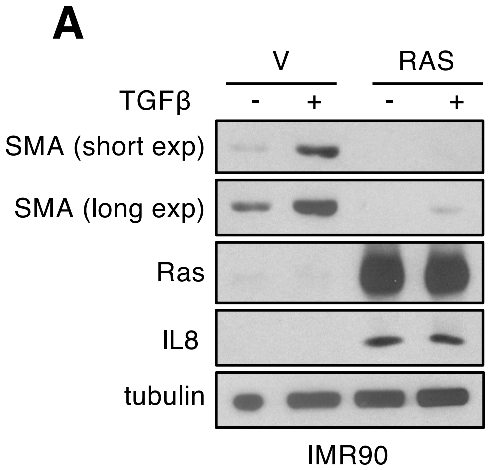

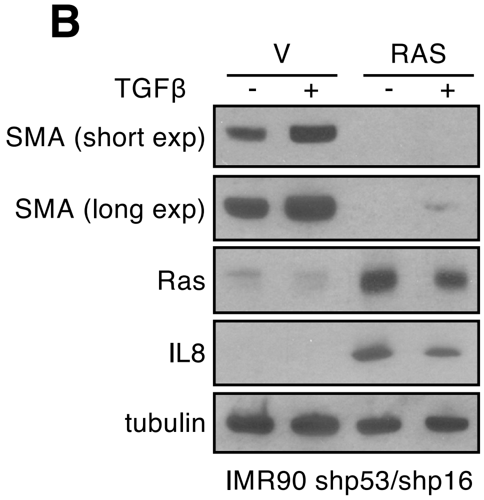

Suppl Figure 6

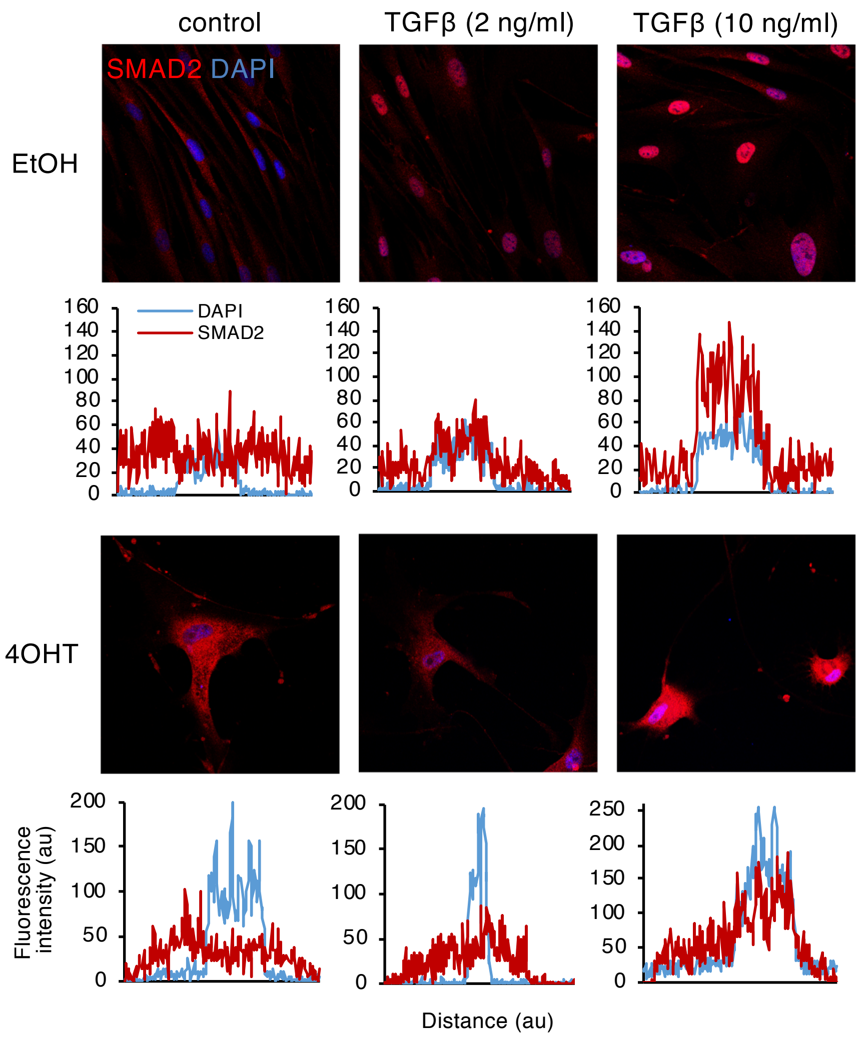

Suppl Figure 7

A

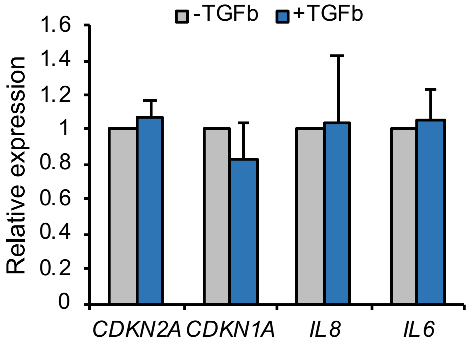

B

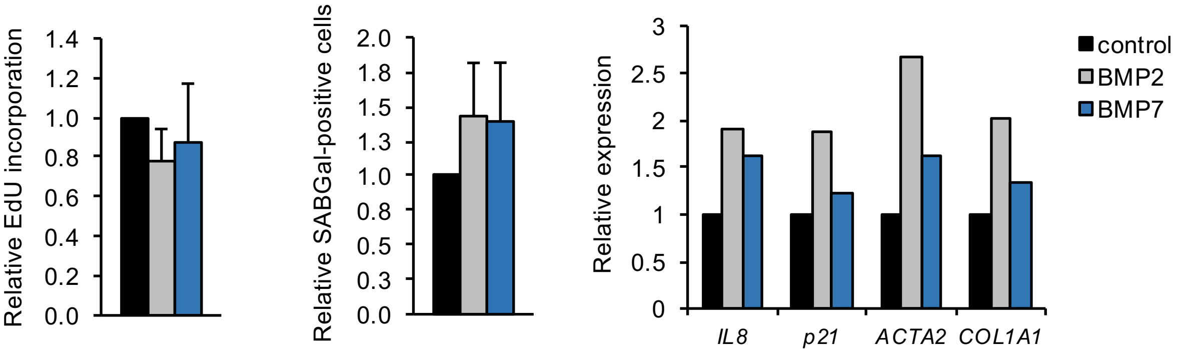

Supp Figure 8

Figure 1A

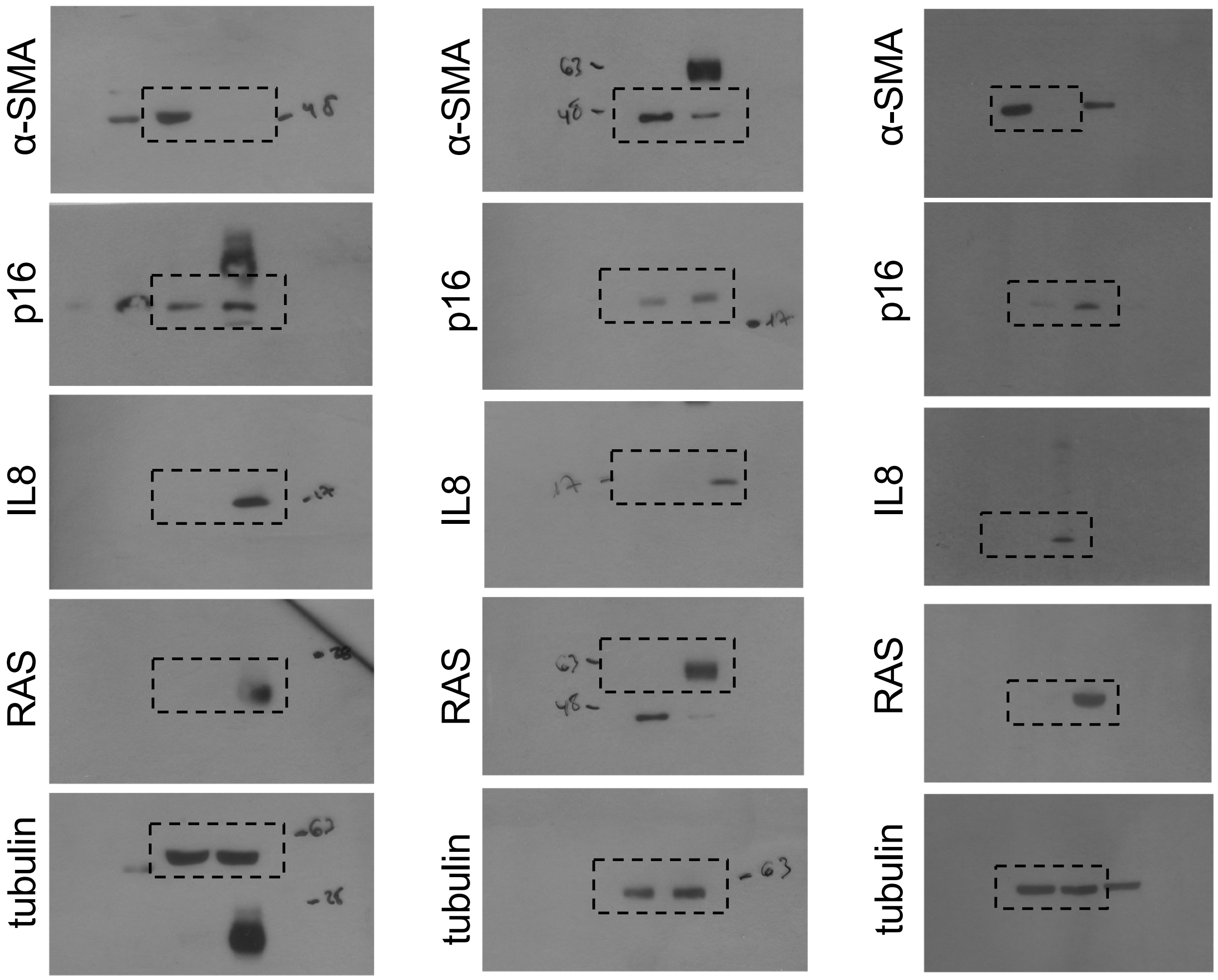

IMR90 RAS IMR90 ER:RAS FF292 ER:RAS

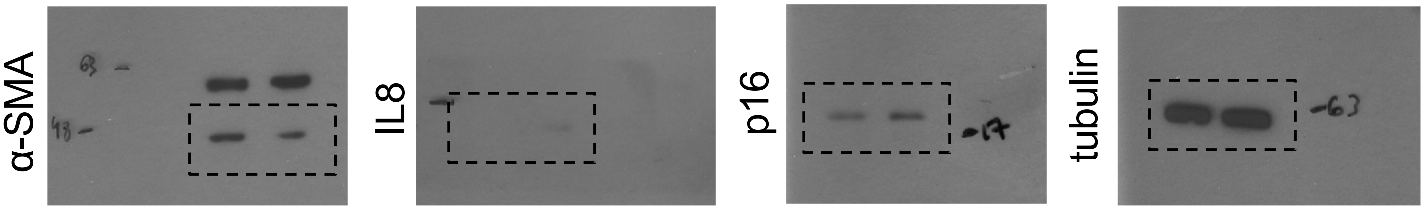

IMR90 Bleo

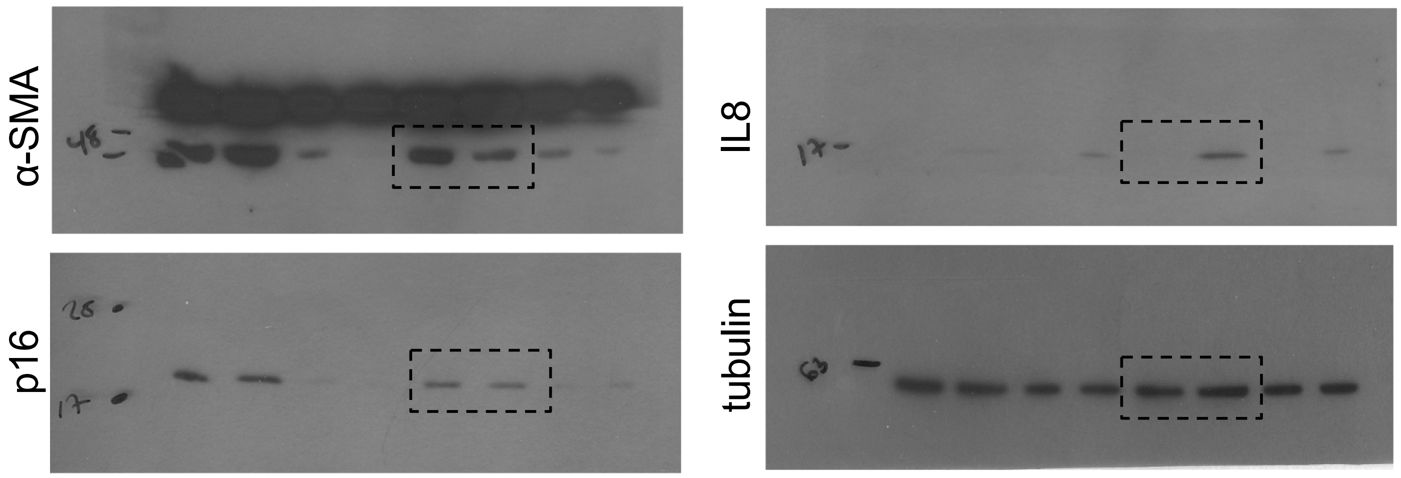

FF292 Bleo

Supp Figure 8 cont

Figure 1C

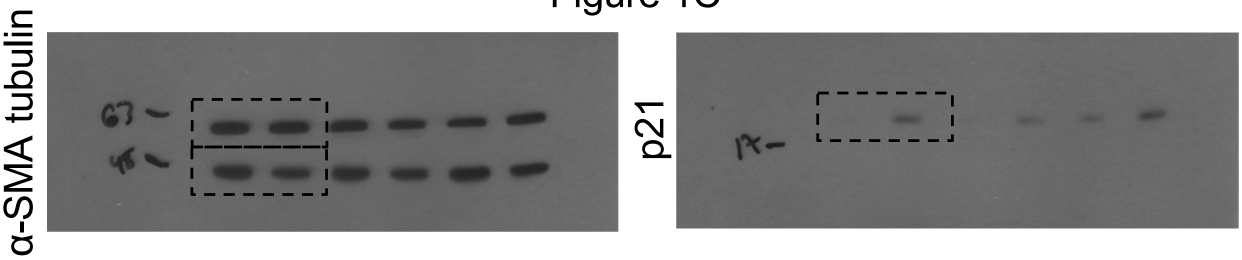

Figure 2A

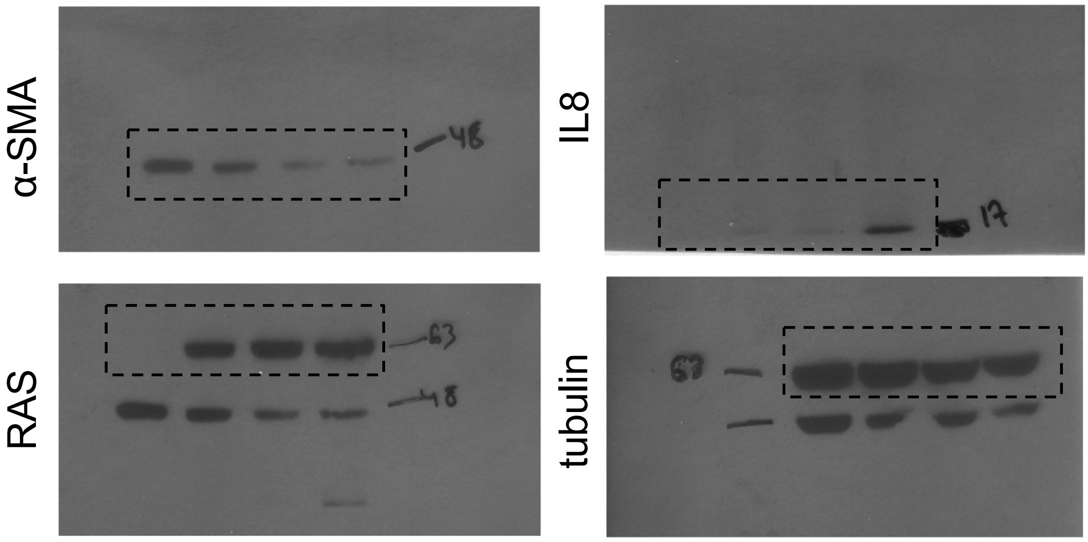

Figure 2G

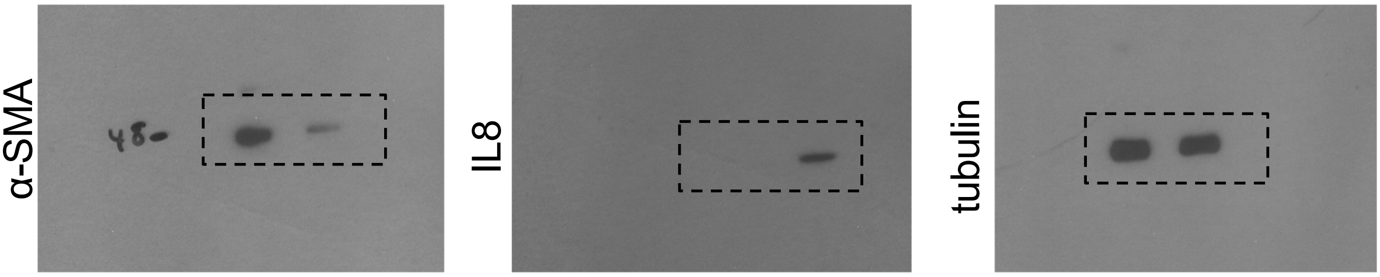

Figure 3C

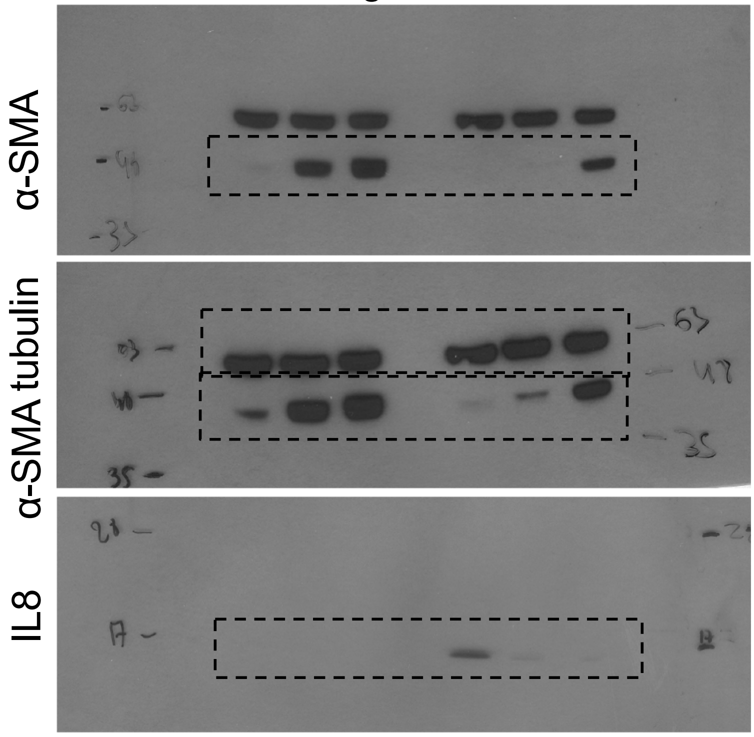

Figure 3F

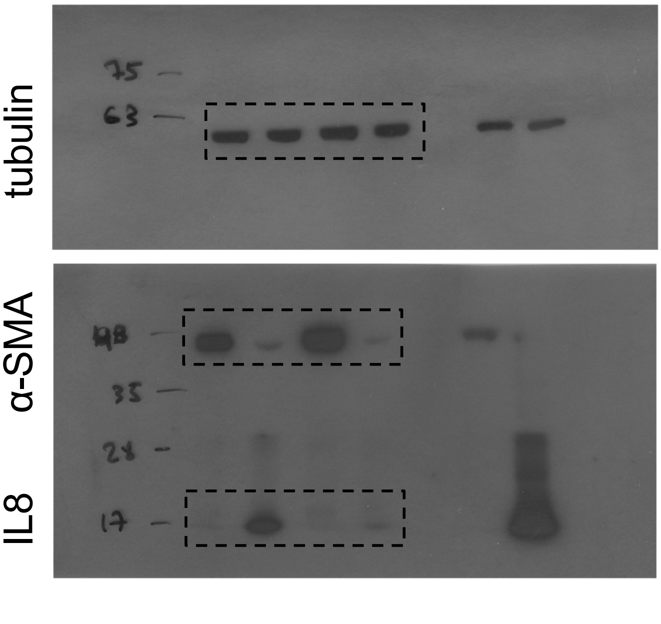

Figure 4D

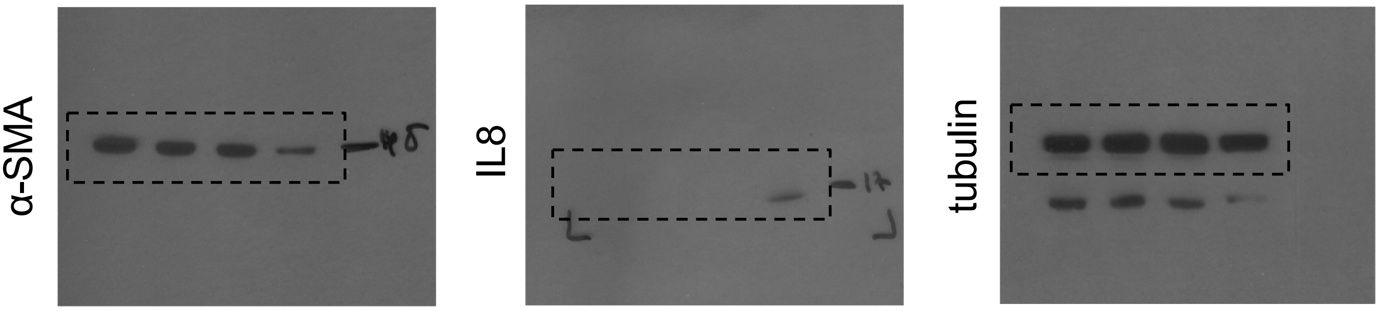

Figure 5A

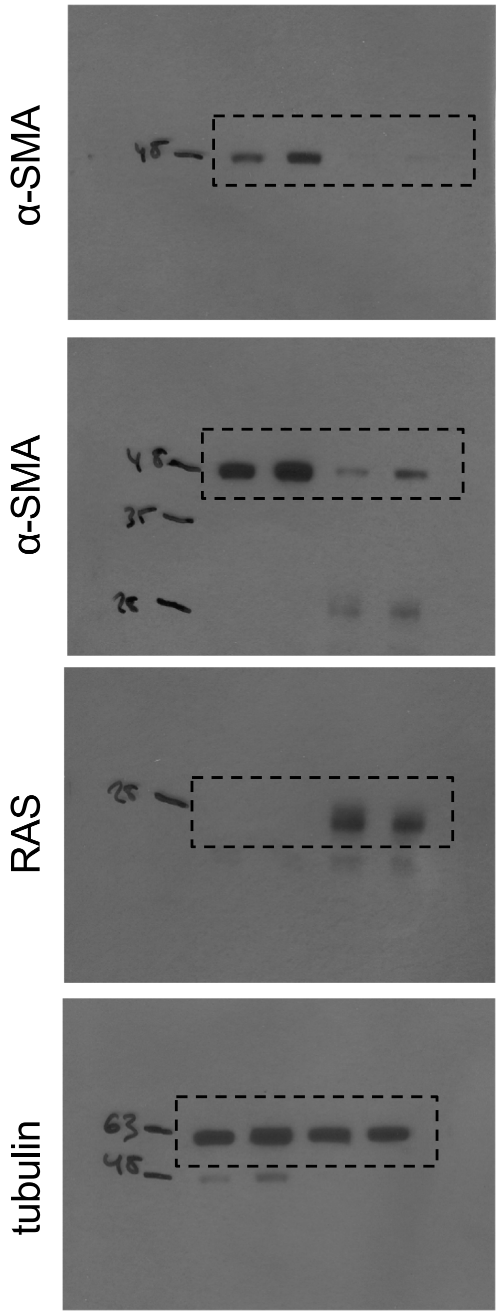

Figure 5F

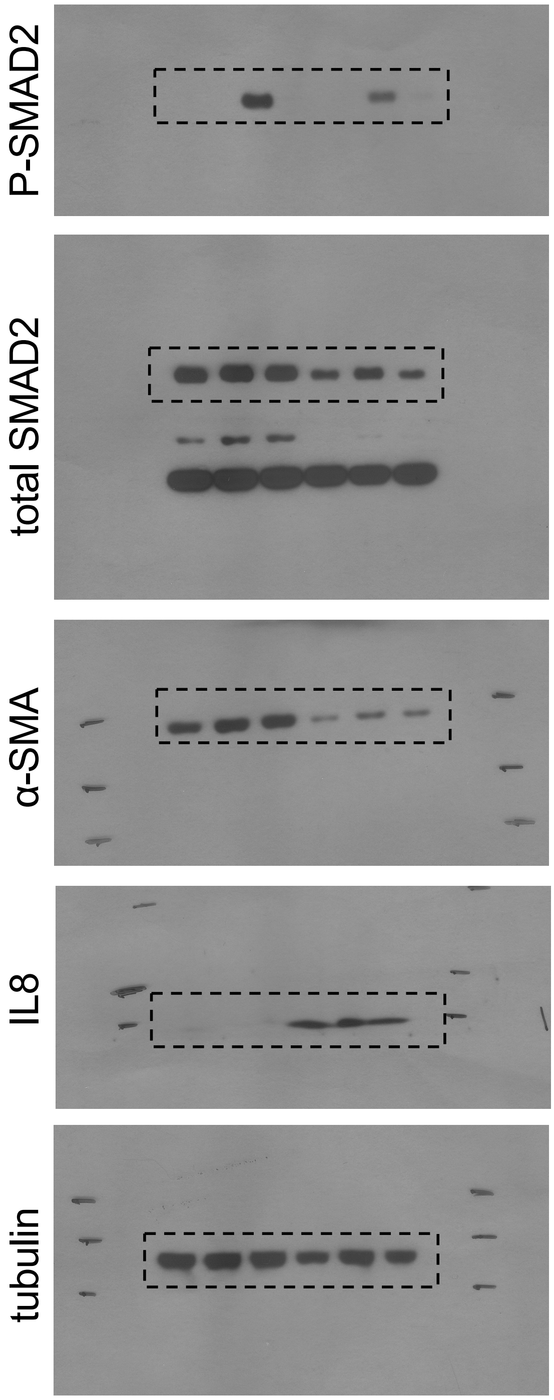

Supp Figure 1C

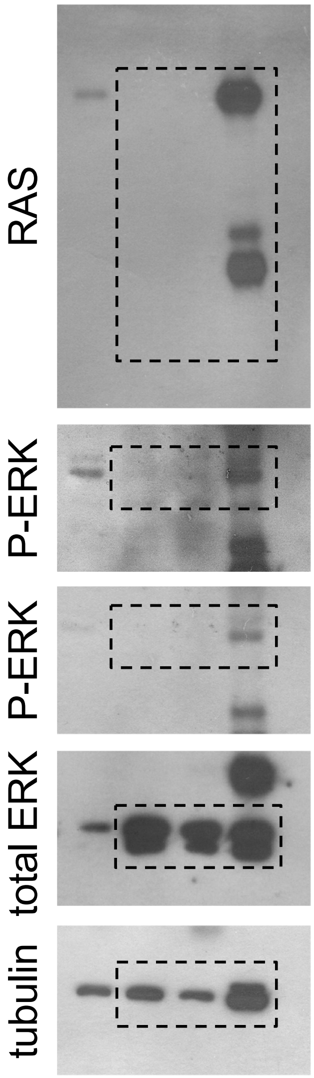

Supp Figure 8 cont

Supp Figure 2A

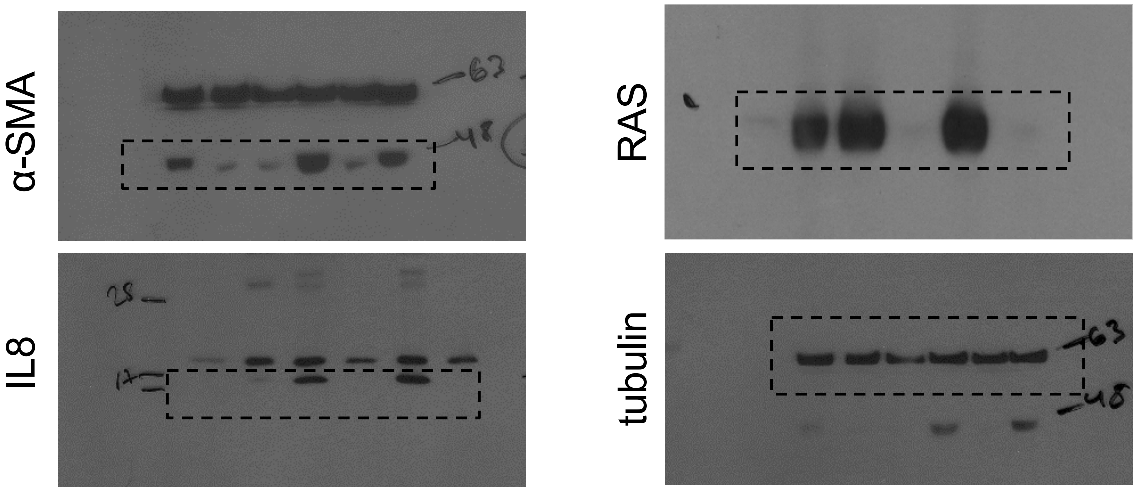

Supp Figure 2B

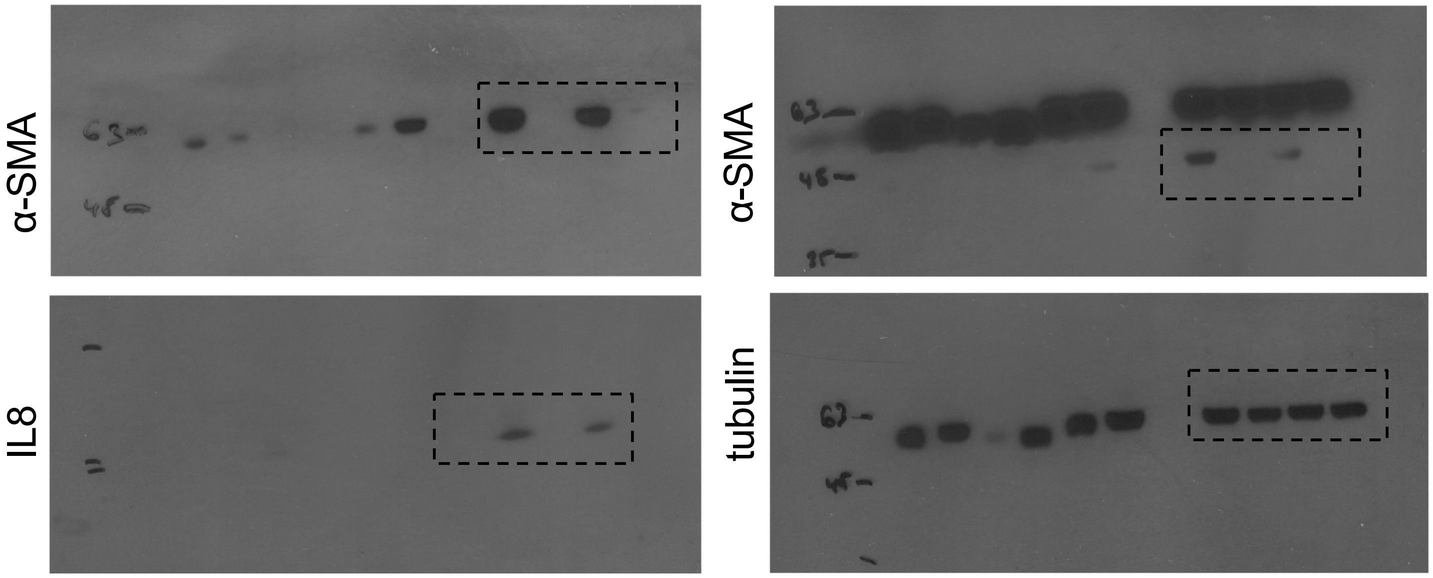

Supp Figure 2D

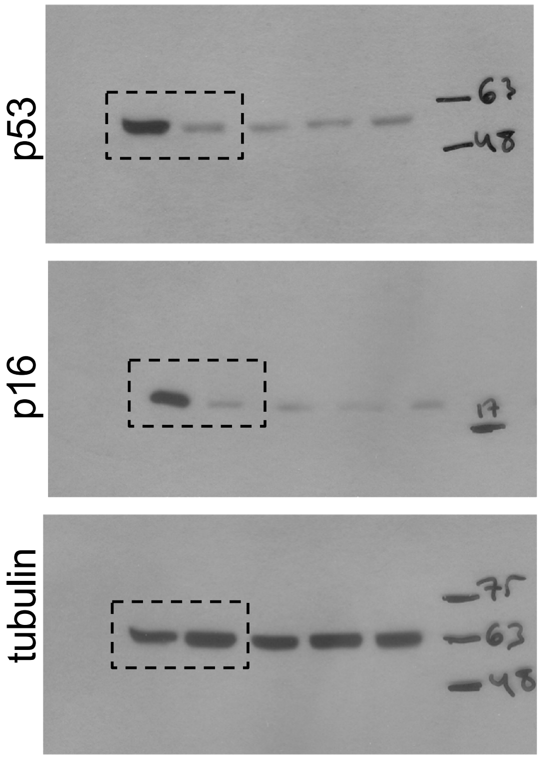

Supp Figure 2E

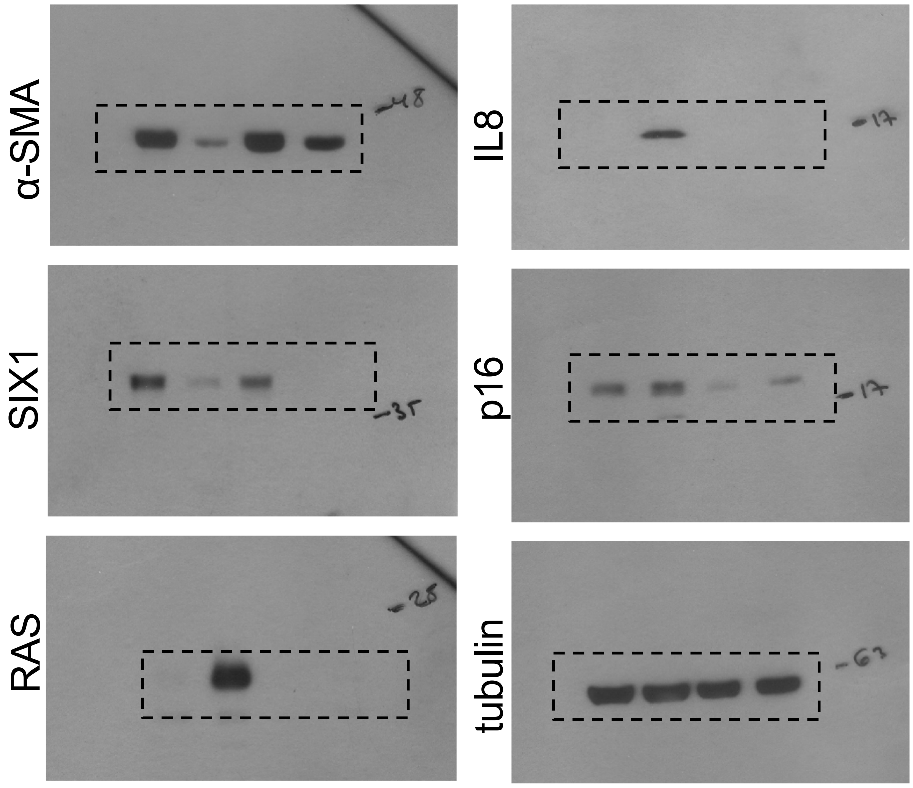

Supp Figure 8 cont

Supp Figure 3C

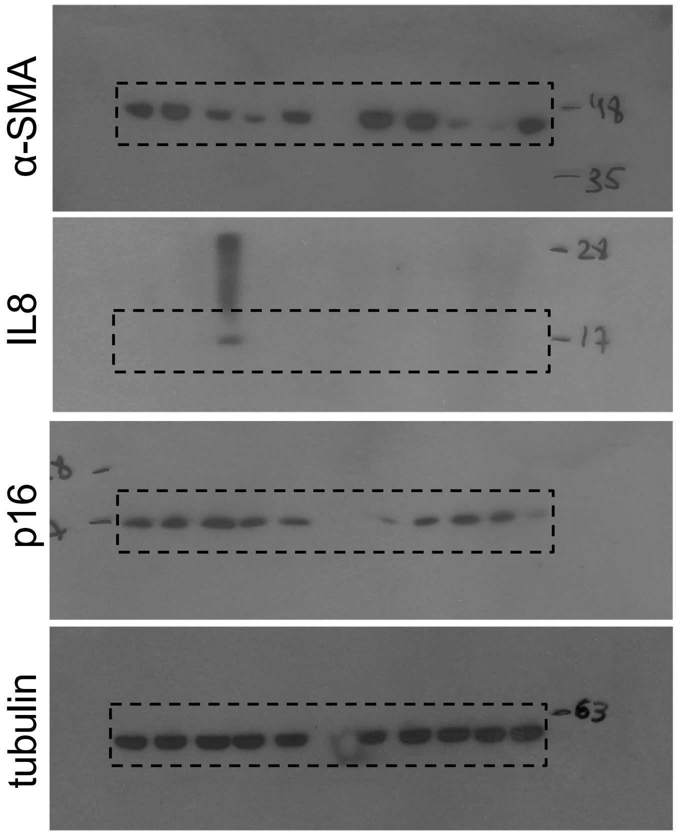

Supp Figure 3D

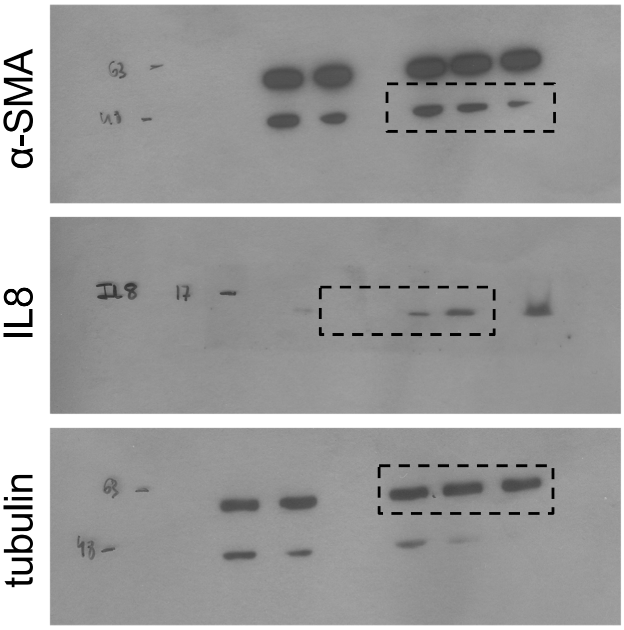

Supp Figure 3E

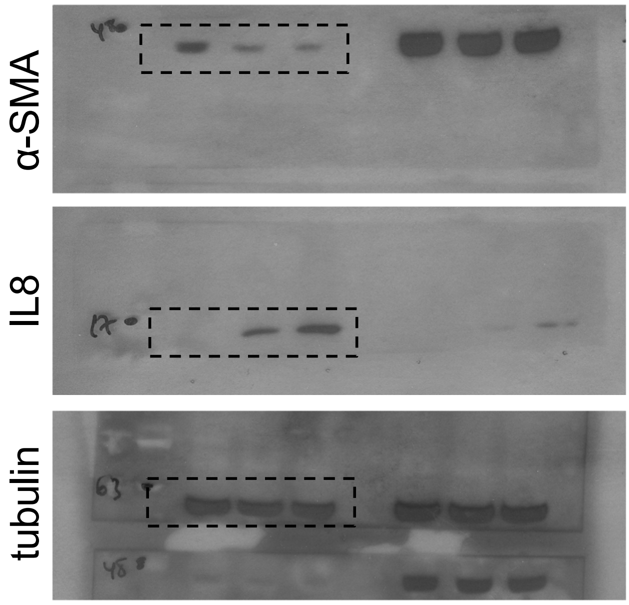

Supp Figure 4A

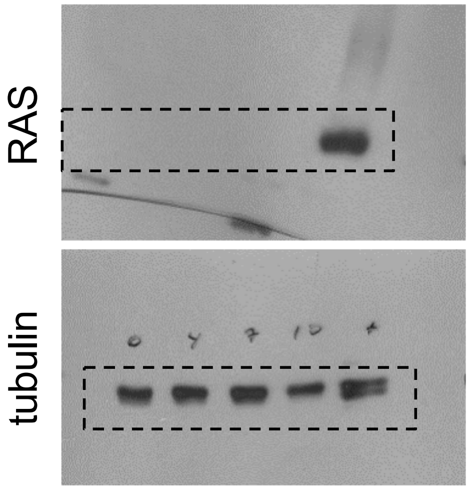

Supp Figure 8 cont

Supp Figure 5A

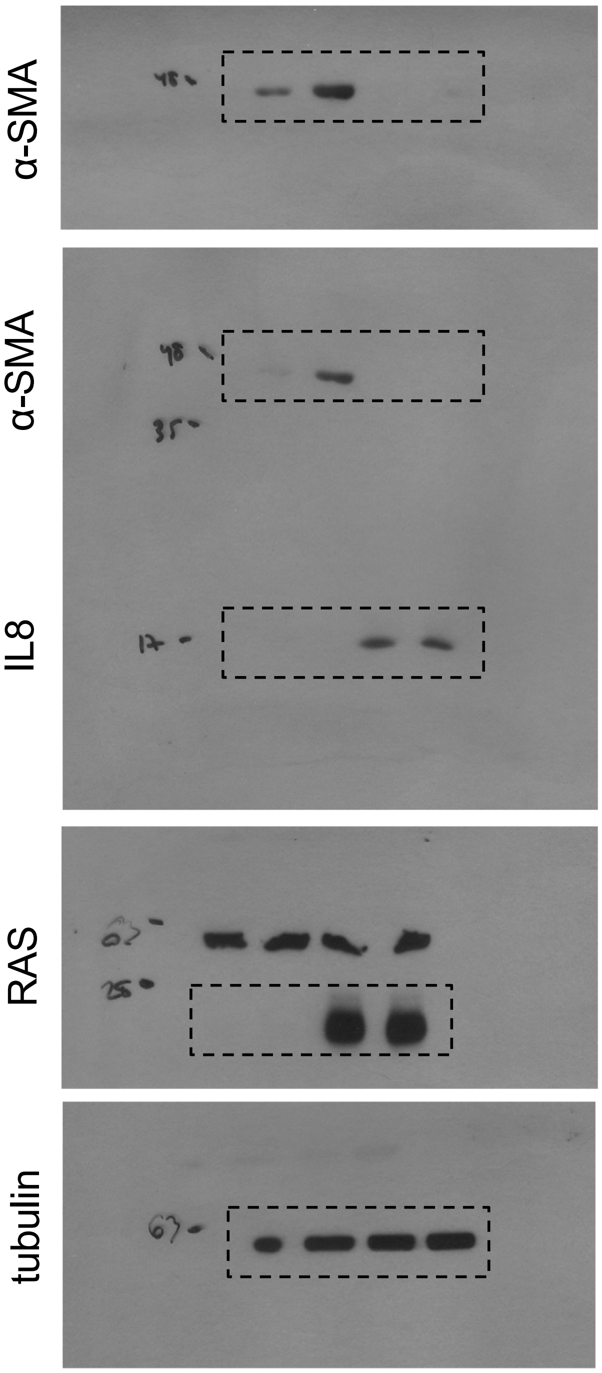

Supp Figure 5B

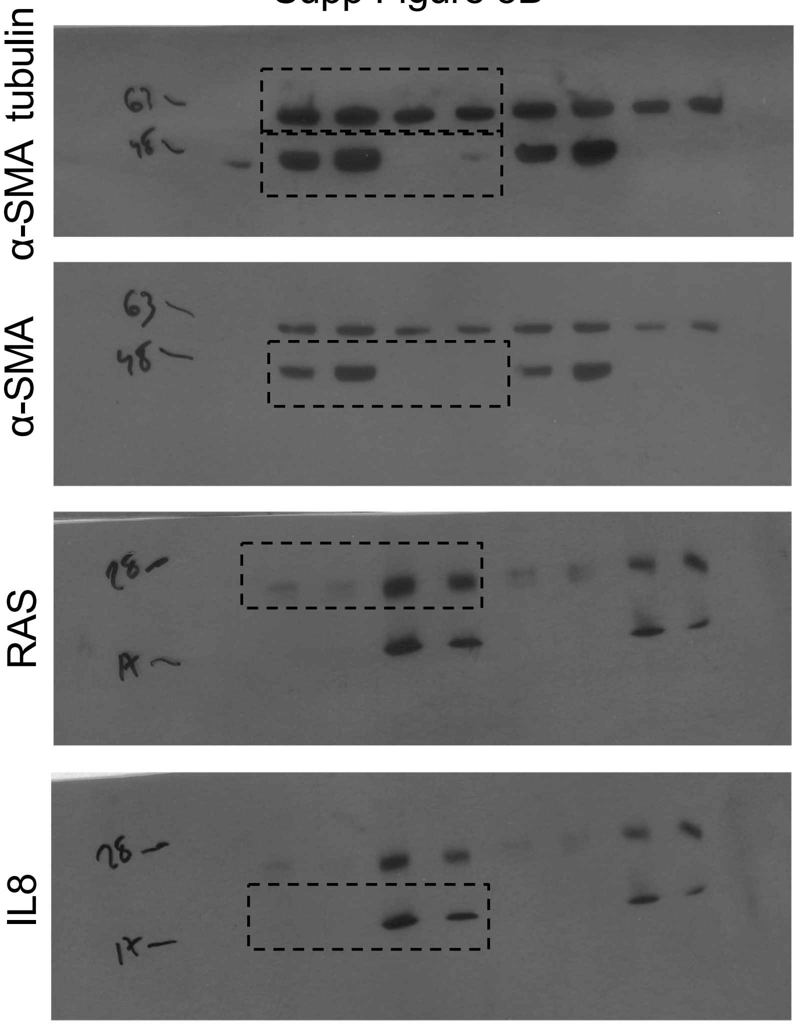

Supplement: Supplementary file 1 — Fig S1‐S8 [file ACEL-21-e13580-s003.pdf]
