# Supplementary material for: Dynamic regulation of myofibroblast phenotype in cellular senescence
Source: Aging Cell. 2022 Mar 9;21(4):e13580. doi: 10.1111/acel.13580 (PMC9009235; doi:10.1111/acel.13580)
Supplement: Supplementary file 2 — Table S1 [file ACEL-21-e13580-s001.pdf]

Supp Table 1

Primers for QPCR (5' to 3')

Gene	Forward primer	Reverse primer
Human *ACTA2*	CCGACCGAATGCAGAAGGA	ACAGAGTATTTGCGCTCCGAA
Human *BMP2*	TCCATGTGGACGCTCTTTCA	GGTCGACCTTTAGGAGACCG
Human *BMP4*	CCACAGCACTGGTCTTGAGT	CCAGATGTTCTTCGTGGTGGA
Human *BMP7*	GTGTGCCTTCCCTCTGAACT	CGTTTCCGGGTTGATGAAGTG
Human *COL1A1*	CAGCCGCTTCACCTACAGC	TTTTGTATTCAATCACTGTCTTGCC
Human *COL1A2*	GGCCCTCAAGGTTTCCAAGG	CACCCTGTGGTCCAACAACTC
Human *COL3A1*	AATCAGGTAGACCCGGACGA	TTCGTCCATCGAAGCCTCTG
Human *GDF15*	CCTGCAGTCCGGATACTCAC	CCCGAGAGATACGCAGGTG
Human *HEY1*	CCGCTGATAGGTTAGGTCTCATTTG	TCTTTGTGTTGCTGGGGCTG
Human *ID4*	ACTGCGCTCAACACCGACCC	GGCCGCACACCTGGACAGC
Human *IL6*	GATGAGTACAAAAGTCCTGATCCA	CTGCAGCCACTGGTTCTGT
Human *IL8*	AGACAGCAGAGCACACAAGC	ATGGTTCCTTCCGGTGGT
IKBalpha mut	GCCGGCCTGGACGCCAT	GGCACCTCCTGCGGCTC
Human *TGFBI*	CCACCATCACCAACAACATCCAG	GCCGTTACCTTCAAGCATCGTG
Human *TGFB1I1*	CATGTCTCAGTTCCCATCTAGCA	GGAAGGGAGGCTGGGTCTTT
Human *TGFB2*	ATTTGCAGGTATTGATGGCACC	AGATGTGGGGTCTTCCCACT
Human *TGFB3*	TGCGTGAGTGGCTGTTGAGAAG	CCATTGGGCTGAAAGGTGTGAC
Mouse *Acta2*	CGTACAACTGGTATTGTGCTGGAC	TGATGTCACGGACAATCTCACGCT
Mouse *Col1a1*	TTCTCCTGGCAAAGACGGACTCAA	AGGAAGCTGAAGTCATAACCGCCA
Mouse *Il6*	GATGGATGCTACCAAACTGGAT	CCAGGTAGCTATGGTACTCCAGA
GFP	CTACCCCGACCACATGAAGC	AAGAAGATGGTGCGCTCCTG
18S rRNA	CCAGTAAGTGCGGGTCATAAGC	CCTCACTAAACCATCCAATCGG

Primary antibodies

Protein	Reference and source	Dilution
Actin	A5441, Sigma	1:10000 (WB)
Collagen I	ab34710, Abcam	1:1000 (IF)
ERK	sc93, Santa Cruz	1:500
Phospho-ERK	sc7383, Santa Cruz	1:500
Fibronectin 1	F3648, Sigma	1:400 (IF)
IL8	MAB208, R&D	1:500 (WB)
IL8	511406, Biolegend (FITC)	1:1000 (IF)
Ki67	RM-9106, Neomarkers	1:500 (IF)
Pan-RAS	sc-166691, Santa Cruz	1:200 (WB)
p16	JC8, CRUK	1:50 (WB)
p21	sc-397-G, Santa Cruz	1:500 (WB)
p53	sc-126, Santa Cruz	1:500 (WB)
SIX1	HPA001893, Sigma	1:500 (WB)
α-SMA	M0851, Dako	1:500 (WB)
α-SMA	C6198, Sigma (Cy3)	1:750 (IF)
α-SMA	A5228, Sigma	1:500 (IF)
SMAD2	5339, Cell Signaling	1:500 (WB)<br>1:50 (IF)
Phospho-SMAD2	3108, Cell Signaling	1:1000 (WB)
Tubulin	T9026, Sigma	1:10000 (WB)

WB: Western Blot, IF: immunofluorescence

Secondary antibodies

Protein	Reference and source	Dilution
Goat anti-mouse HRP	P0447, Dako	1:5000
Monkey anti-rabbit HRP	NA934V, GE Healthcare	1:5000
Rabbit anti-goat HRP	P0160, Dako	1:5000
Donkey anti-rabbit-Alexa 488	A21206, Invitrogen	1:500
Donkey anti-rabbit-Alexa 555	A31572, Invitrogen	1:500
Donkey anti-mouse-Alexa 546	A10036, Invitrogen	1:500
Donkey anti-mouse-Alexa 488	A21202, Invitrogen	1:500
