# Supplementary material for: Dynamic regulation of myofibroblast phenotype in cellular senescence
Source: Aging Cell. 2022 Mar 9;21(4):e13580. doi: 10.1111/acel.13580 (PMC9009235; doi:10.1111/acel.13580)
Supplement: Supplementary file 3 — Table S2 [file ACEL-21-e13580-s002.pdf]

Supp Table 2

UP	Cluster 3<br>12 genes	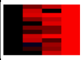	KEGG: Cytokine-cytokine receptor interaction (IL1B, CXCL8, CXCL1, IL1A, CXCL5) q=1.16E-9	KEGG: TNF signalling pathway (IL1B, CXCL1, CXCL5, MMP3) q=0.000003247	Wikipathways: Senescence and autophagy in cancer (CXCL8, CXCL1, IL1B, IL1A, IL24) q=1.393E-7	Reactome: Signalling by GPCR (CXCL5, CXCL1, CXCL8, C3, CCL7, MMP3) q=0.000037	Biocarta: NFkB signalling (IL1A) q=0.01	Jensen compartments: Extracellular space (IL1A, CXCL1, IL1B, MMP3, IL24, CCL7, C3, IL13RA2, CXCL5) q=0.000001063	
	Cluster 11<br>19 genes	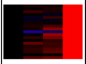	KEGG: Cytokine-cytokine receptor interaction (IL6, IL11, CXCL10, CCL20, CXCL3, TNFSF15) q=0.0000054	KEGG: TNF signalling pathway (IL6, CXCL10, CCL20, CXCL3) q=0.00007088	Wikipathways: Senescence and autophagy in cancer (IL6, TNFSF15, SERPINB2) q=0.003268	Reactome: G alpha signalling events (CXCL10, CCL20, CXCL3, RGS18) q=0.004354			
	Cluster 12<br>8 genes	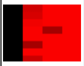	GO Cellular component: ER-Golgi (AREG) q=0.03	Wikipathways: Matrix metalloproteinases (MMP1) q=0.02	Reactome: Collagen degradation (MMP1) q=0.05				
	Cluster 16<br>7 genes	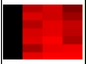	KEGG: ECM-receptor interaction (ITGA10) q=0.05	GO Molecular function: Protein binding involved in cell-matrix adhesion (ITGA10) q=0.01	KEGG: Notch signaling pathway (DLL4) q=0.05	Wikipathways: Notch regulation of human endothelial cell calcification (DLL4) q=0.05			
	Cluster 17<br>12 genes	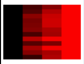	Wikipathways: Senescence and autophagy in cancer (PLAT, BMP2) q=0.01698	Wikipathways: Quercetin and NFkB/AP-1 induced cell apoptosis (PTGS2) q=0.04	Reactome: Signalling by BMP (GREM2, BMP2) q=0.002653	Reactome: Dissolution of fibrin clot (PLAT) q=0.033			
	Cluster 18<br>31 genes	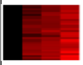	GO Biological process: Mesodermal cell differentiation (ITGA2, HMGA2) q=0.002965	Biocarta: Platelet amyloid precursor protein pathway (PLAU) q=0.03	Biocarta: Fibrinolysis pathway (PLAU) q=0.03	KEGG: Mineral absorption (MT2A, MT1E, MT1L) q=0.001	GO Biological process: Cellular response to ions (MTL1, MT1E, MT2A) q=0.00057		
	Cluster 13<br>16 genes	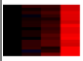	Biocarta: CD40L signaling pathway (TNFAIP3) q=0.01						
	Cluster 10<br>7 genes	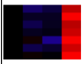	GO Molecular function: BMP binding (GREM1) q=0.01	Reactome: Amino acid transport across the plasma membrane (SLC7A2) q=0.04					
	Cluster 6<br>13 genes	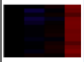	KEGG: Apoptosis (CTSK, ERN1) q=0.01	GO Biological process: Positive regulation of ECM disassembly (PDPN) q=0.05	Wikipathways: Osteoclast signaling (TNFRSF11B, CTSK) q=0.00062	*Wikipathways: TGFb signaling pathway (TGIF1) q=0.1*	Kinase perturbations: TGFBR2 knockout (perturbations) (TNFRSF11B, CTSK, NUAK2, PDE7B) q=0.0018	Reactome: Metal ion transporters (SLC39A14, SLC11A2) q=0.0049	KEGG: Lysosome (CTSK, SLC11A2, ARSG) q=0.0008
	Cluster 14<br>24 genes	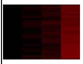	Biocarta: IL6 signaling pathway (CEBPB) q=0.05	Panther: Apoptosis signaling pathway (MCL1, RELB) q=0.04	GO Biological process: Positive regulation of myoblast differentiation (RIPOR2, BTG1) q=0.04	Wikipathways: Myometrial relaxation and contraction pathways (MAFF, ETS2, GJA1) q=0.02			
	Cluster 15<br>29 genes	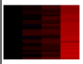	KEGG: TNF signaling pathway (NFKBIA, CCL2, ICAM1, LIF) q=0.0001	Reactome: Anchoring fibril formation (LAMB3, COL7A1) q=0.01	GO Biological process: Positive regulation of cellular extravasation (RIPOR2, ICAM1) q=0.01	Wikipathways: Complement and coagulation cascades (PLAUR, BDKRB1) q=0.05			
	Cluster 19<br>30 genes	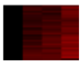	KEGG: TNF signaling pathway (MAPK13, IKBKB, VEGFC) q=0.01	Wikipathways: Heart development (VEGFC, SMAD1) q=0.05	Biocarta: Keratinocyte differentiation (MAPK13, IKBKB) q=0.03	*Panther: TGFb signaling pathway (MAPK13, SMAD1) q=0.09*			
	Cluster 20<br>19 genes	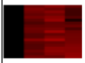	*Reactome: Integrin cell surface interactions (ITGA6, COL13A1) q=0.08*	*Reactome: Collagen formation (ITGA6, COL13A1) q=0.08*					

DOWN	Cluster 4<br>9 genes	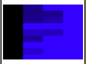	GO Biological Process: Muscle hypertrophy (HTR2B) q=0.04	GO Biological Process: Blood vessel diameter (HTR2B) q=0.04	Mammalian phenotype: Cardiovascular system physiology (TNNT2, HTR2B) q=0.04	Mammalian phenotype: Abnormal heart development (TNNT2, HTR2B) q=0.03			
	Cluster 8<br>41 genes	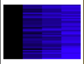	*KEGG: Vascular smooth muscle contraction (GUCY1B3, ADRA1B, ACTA2) q=0.07*	KEGG: Myometrial relaxation and contraction (OXTR, CORIN, ACTA2, IGFBP3) q=0.005	*KEGG: Focal adhesion-PI3K-AKT-mTOR (PDGFD, COL11A1, FGF9) q=0.14*	*GO Molecular function: Type II transforming growth factor b receptor binding (BAMBI) q=0.09*	*Panther: TGFb signalling (GDF5, BAMBI) q=0.13*		
	Cluster 7<br>59 genes	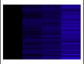	*Reactome: Smooth muscle contraction (ACTG2, TPM2) q=0.8*	*Reactome: Extracellular matrix organization (SDC2, NTN4, COL8A2, BMP4) q=0.6*	GO Biological Process: Cell differentiation involved in metanephros development (OSR1, BMP4) q=0.04	GO Biological Process: Cell migration involved in heart development (PDGFRB, SNAI2) q=0.03	GO Biological Process: Cardiac muscle cell differentiation (BMP4, EFNB2) q=0.04	*Reactome: TGFb signalling (ID2, BMP4) q=0.8*	
	Cluster 9<br>30 genes	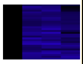	GO Biological Process: Positive regulation of smooth muscle cell proliferation (STAT1, IL6R, HMOX1) q=0.00579	Reactome: Extracellular matrix organization (MFAP4, FBN2, TGFB2, COL14A1, ADAMTS5, ADAMTS1, ADAMTS8, VCAM1) q=1.1E-6	GO Biological Process: Extracellular matrix organization (TGFB2, COL14A1, ADAMTS5, FBN2, VCAM1) q=0.005	GO Biological Process: Embryo development ending in birth or egg hatching (TGFB2, STOX2) q=0.02	Wikipathways: Endochondral ossification (TGFB2, STAT2, ADAMTS5, ADAMTS1) q=0.00009818		
	Cluster 2<br>44 genes	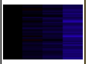	GO Cellular component: Cytoskeleton (CNN2, CNN1, MARCKS, CNN3, CDC42EP3, MTCL1, LMOD1) q=0.002	GO Cellular component: Focal adhesion (CNN2, CNN1, MARCKS, CNN3, LIMS2, FHL2, TES) q=0.0005	GO Cellular component: Rough endoplasmic reticulum (TRAM2, TRAM1) q=0.02				

SASP	ECM	NOTCH	TGFbeta	Coagulation	Differentiation, development	Muscle	Metals	Other
